# Differential patterns of ophiostomatoid fungal communities associated with three sympatric *Tomicus* species infesting pines in south-western China, with a description of four new species

**DOI:** 10.3897/mycokeys.50.32653

**Published:** 2019-04-09

**Authors:** Hui Min Wang, Zheng Wang, Fu Liu, Cheng Xu Wu, Su Fang Zhang, Xiang Bo Kong, Cony Decock, Quan Lu, Zhen Zhang

**Affiliations:** 1 Key Laboratory of Forest Protection, National Forestry and Grassland Administration; Research Institute of Forest Ecology, Environment and Protection, Chinese Academy of Forestry, Beijing 100091, China Research Institute of Forest Ecology, Environment and Protection, Chinese Academy of Forestry Beijing China; 2 Mycothèque de l’Université Catholique de Louvain (BCCM/MUCL), Earth and Life Institute, Microbiology, B-1348 Louvain-la-Neuve, Belgium Mycothèque de l’Université Catholique de Louvain Louvain-la-Neuve Belgium

**Keywords:** *
Esteya vermicola
*, *
Graphilbum
*, *
Leptographium
*, *
Ophiostoma
*, species-specific association, *
Sporothrix
*, taxonomy

## Abstract

Bark beetles and their associated fungi, which cause forest decline and sometimes high mortality in large areas around the world, are of increasing concern in terms of forest health. Three *Tomicus* spp. (*T.brevipilosus*, *T.minor* and *T.yunnanensis*) infect branches and trunks of *Pinusyunnanensis* and *P.kesiya* in Yunnan Province, in south-western China. *Tomicus* spp. are well known as vectors of ophiostomatoid fungi and their co-occurrence could result in serious ecological and economic impact on local forest ecosystems. Nonetheless, knowledge about their diversity, ecology, including pathogenicity and potential economic importance is still quite rudimentary. Therefore, an extensive survey of ophiostomatoid fungi associated with these *Tomicus* species infesting *P.yunnanensis* and *P.kesiya* was carried out in Yunnan. Seven hundred and seventy-two strains of ophiostomatoid fungi were isolated from the adult beetles and their galleries. The strains were identified based on comparisons of multiple DNA sequences, including the nuclear ribosomal large subunit (LSU) region, the internal transcribed spacer regions 1 and 2, together with the intervening 5.8S gene (ITS) and the partial genes of β-tubulin (*TUB2*), elongation factor 1α (*TEF1-α*) and calmodulin (*CAL*). Phylogenetic analyses were performed using maximum parsimony (MP) as well as maximum likelihood (ML). Combinations of culture features, morphological characters and temperature-dependent growth rates were also employed for species identification. Eleven species belonging to five genera were identified. These included six known species, *Esteyavermicola*, *Leptographiumyunnanense*, *Ophiostomabrevipilosi*, *O.canum*, *O.minus* and *O.tingens* and four novel taxa, described as *Graphilbumanningense*, *O.aggregatum*, *Sporothrixpseudoabietina* and *S.macroconidia*. A residual strain was left unidentified as *Ophiostoma* sp. 1. The overall ophiostomatoid community was by far dominated by three species, representing 87.3% of the total isolates; in decreasing order, these were *O.canum*, *O.brevipilosi* and *O.minus*. Furthermore, the ophiostomatoid community of each beetle, although harbouring a diversity of ophiostomatoid species, was differentially dominated by a single fungal species; *Ophiostomacanum* was preferentially associated with and dominated the ophiostomatoid community of *T.minor*, whereas *O.brevipilosi* and *O.minus* were exclusively associated with and dominated the ophiostomatoid communities of *T.brevipilosus* and *T.yunnanensis*, respectively. Eight additional species, representing the remaining 12.7% of the total isolates, were marginal or sporadic. These results suggested that sympatric *Tomicus* populations are dominated by distinct species showing some level of specificity or even exclusivity.

## Introduction

Associations between insects and microorganisms are increasingly recognised as one of the major issues in forest ecology and forest health around the world (Wingfield et al. 2016). Many bark beetles are well known as tree pests causing various levels of tree mortality and forest decline in large areas of the world, mostly in temperate areas (Jankowiak 2006, Wingfield et al. 2017). These bark beetles are well known vectors of variably pathogenic fungi, forming symbiosis-like relationships (Six 2003, Lu et al. 2009).

The pine shoot beetles, *Tomicus* Latreille (syn. *Blastophagus* Eichhoff, *Myelophilus* Eichhoff, Scolytidae, Coleoptera), are destructive insects with a range spanning the Eurasian pine forests, seriously affecting tree growth and causing a great threat to the forest ecosystems (Kirkendall et al. 2008, Lieutier et al. 2015). Currently, eight species are recorded worldwide, i.e. *T.armandii* Li and Zhang (Li et al. 2010), *T.brevipilosus* Eggers, *T.destruens* Wollaston, *T.minor* Hartig, *T.pilifer* Spessivtsev, *T.piniperda* L., *T.puellus* Reitter, and *T.yunnanensis* Kirkendall and Faccoli (Kirkendall et al. 2008). They all occur in China except *T.destruens* and five of them, viz. *T.armandii*, *T.brevipilosus*, *T.minor*, *T.pilifer* and *T.yunnanensis*, are sympatric in forests of the Yunnan Province (Li et al. 1997, 2010, Kirkendall et al. 2008; Ye 2011). *Tomicusbrevipilosus*, *T.minor* and *T.yunnanensis* have overlapping geographical distribution, host range and infection periods. They aggregately infect branches and trunks of two indigenous pines, *Pinusyunnanensis* and *P.kesiya* (Li et al. 1997, 2006, Chen et al. 2009, 2010, Lu et al. 2012, 2014), causing locally extensive tree decline or mortality (Ye and Dang 1986, Ye 1991, 2011). Since the 1980s, damage caused by these bark beetles has resulted in losses of more than 93,000 m^3^ of pinewood (Ji et al. 2007).

Generally, two or three pine shoot beetles co-occur underneath the bark or in shoots of a single host tree, either simultaneously but with spatially isolated galleries or successively, during differential infesting peaks. Spatial and chorological differentiation would reduce competition between beetles, but their co-occurrence also could enhance cooperation (Lu et al. 2012, Chen et al. 2015). *Tomicusyunnanensis* is considered to be the most aggressive species in Yunnan, causing primary infestations of healthy *P.yunnanensis* trees and eventually tree death (Ye and Lieutier 1997, Kirkendall et al. 2008, Chen et al. 2010, 2015, Lu et al. 2014). Although *T.brevipilosus* is able to infect healthy trees, it preferably colonises trunks already infested by *T.yunnanensis* or both *T.yunnanensis* and *T.minor* (Chen et al. 2010, 2015). *Tomicusminor* is often regarded as a secondary, opportunist species infesting trees already weakened by *T.yunnanensis* or/and *T.brevipilosus* (Ye and Ding 1999, Lieutier et al. 2003, Chen et al. 2009).

Pine shoot beetles such as *T.piniperda*, *T.minor* and *T.destruens* are commonly associated with ophiostomatoid fungi (Masuya et al. 1999, Kim et al. 2005, Jankowiak 2006, 2008). Fifteen ophiostomatoid fungi were reported associated with *T.piniperda* in Europe (Mathiesen 1950, Lieutier et al. 1989, Gibbs and Inman 1991, Solheim and Långström 1991, Jankowiak 2006, Jankowiak and Bilański 2007) and 11 were documented in eastern Asia (Japan and Korea) (Masuya et al. 1999, Kim et al. 2005). *Ophiostomaminus* was shown to be the dominant species associated with *T.piniperda* in Europe and Japan (Mathiesen 1950, Lieutier et al. 1989, Gibbs and Inman 1991, Masuya et al. 1999, Jankowiak 2006). *Leptographiumwingfieldii* was shown to be the strongest pathogenic one (Gibbs and Inman 1991) in Europe. *Tomicusminor* also infests various pines in Europe and Asia. Fifteen (Mathiesen-Käärik 1953, Masuya et al. 1999, Jankowiak 2008) and 11 (Masuya et al. 1999) ophiostomatoid species have been reported to be associated with this beetle species in Europe and Japan, respectively. *Ophiostomacanum* was recorded as a frequent/dominant species in association with *T.minor*, both in Europe and Japan (Mathiesen 1950, 1951, Rennerfelt 1950, Francke-Grosmann 1952, Masuya et al. 1999) but seems to represent a weak pathogen to *P.sylvestris* (Solheim et al. 2001). Additionally, six ophiostomatoid fungi were documented associated with *T.destruens* in Europe (Lieutier 2002, Sabbatini Peverieri et al. 2006, Ben Jamaa et al. 2007).

Despite the fact that *Tomicus* spp. have caused serious losses to forest ecosystems in south-western China, there are no systematic studies of their ophiostomatoid associates but only a few sporadic reports. So far, nine ophiostomatoid species have been reported as being associated with *Tomicus* spp. in Yunnan. Six species (*Leptographiumyunnanense*, *Ophiostomaips*, *O.minus*, *O.quercus*, *S.abietina* and *S.nebularis*) were recorded to be associated with *T.yunnanensis* (Ye et al. 2000, Zhou et al. 2000, 2013, Chang et al. 2017). Two species (*Graphilbumfragrans* and *O.tingens*) were recorded as being associated with *T.minor* (Zhou et al. 2013, Pan et al. 2017), whereas only a single species (*O.brevipilosi*) was recorded as being associated with *T.brevipilosus* (Chang et al. 2017). Amongst them, *L.yunnanense* was the first species newly described from the area (Zhou et al. 2000) and is likely the most virulent one (Liao and Ye 2004, Gao et al. 2017). Until now, the relative abundance with which these fungi occur, their host (pine and beetle) relationships, and their pathogenicity remain unknown.

The symbiosis between bark beetles and ophiostomatoid fungi enhances their pathogenicity. The fitness of bark beetle populations may depend in part on the degree of the fungal partners’ pathogenicity and the resulting weakening of the tree (Christiansen et al. 1987, Kirisits 2004, Linnakoski et al. 2012), although this has been questioned by some (Six and Wingfield 2011). Therefore, the question remains whether there is any link between the differential aggression of the pine shoot beetles and the differential virulence of their fungal associates, especially in circumstances where various beetle species co-exist.

The aim of this study was to describe the diversity of ophiostomatoid fungal communities associated with three pine shoot beetles and their galleries infesting *P.yunnanensis* and *P.kesiya* in forest ecosystems of Yunnan Province. We also analysed the degree of beetle/ophiostomatoid fungi specificity. Such studies will enable us to understand the aggressive nature of the beetles and the pathogenicity of the associated fungi and the interactions, ultimately helping to address the current situation of ceaseless outbreaks and rapid expansion of the pests.

## Materials and methods

### Sample collection and fungus isolation

**Figure 1.**
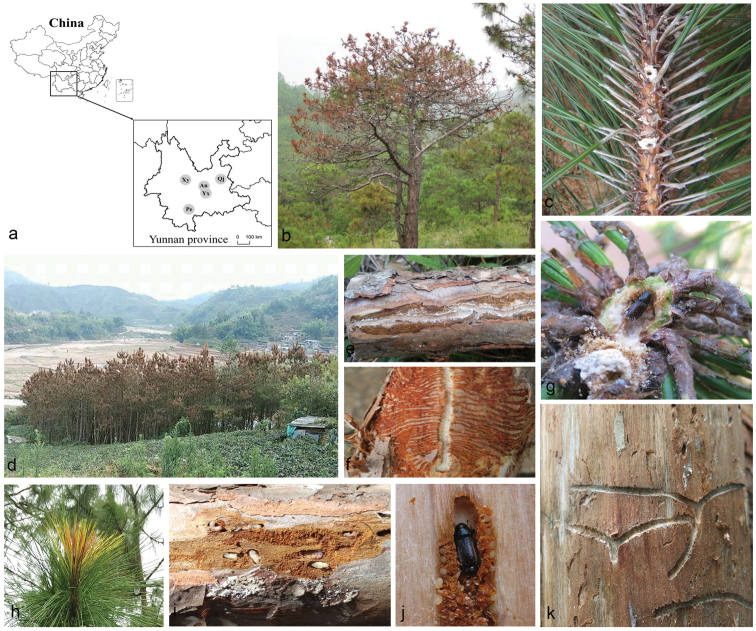
**A** Map showing the 11 species of ophiostomatoid fungi detected from Yunnan Province, China **B, D** disease symptoms on *Pinusyunnanensis* and *P.kesiya* trees infested by *Tomicus* spp. (*T.yunnanensis*, *T.minor* and *T.brevipilosus*) and ophiostomatoid fungi **C, G, H** exposed branches of *Tomicus* spp. on *P.yunnanensis* and *P.kesiya*** E, F, I–K** galleries of *Tomicus* spp. on *P.yunnanensis* and *P.kesiya*.

Samples of galleries in bark and shoots and adults of *Tomicus* spp. were collected from *P.yunnanensis* and *P.kesiya* at five sites in Yunnan Province (Fig. 1, Table 1) from December 2016 to March 2017. Beetles were placed individually in sterilised Eppendorf tubes and their galleries were placed in sterile envelopes and stored at 4°C until processed within one week.

**Table 1.** Basic information on the sample collection plots in China.

**Location**	**Host**	**Insect vector**	**longitude\latitude**	**altitude(m)**	**No. of examained samples**
Xiangyun,Yunnan	* Pinus yunnanensis *	*Tomicusyunnanensis*, *T.minor*	25°21'25.8"N, 100°51'49"E	2255.4	447
Puer,Yunnan	* P. kesiya *	*T.brevipilosus*, *T.minor*	22°56'36.1"N, 101°14'36.7"E	1400.7	346
Qujing,Yunnan	* P. yunnanensis *	*T.yunnanensis*, *T.minor*, *T.brevipilosus*	25°28'51"N, 103°46'32"E	2068.2	102
Anning,Yunnan	* P. yunnanensis *	*T.yunnanensis*, *T.minor*, *T.brevipilosus*	24°53'32"N, 102°24'23"E	1939.9	138
Yuxi, Yunan	* P. yunnanensis *	*T.yunnanensis*, *T.minor*	24°18'23"N, 102°34'37"E	1908.1	85

Isolations from beetles and their galleries were carried out on 2% malt extract agar (MEA: 20 g Biolab malt extract, 20 g Biolab agar and 1 000 ml deionised water) with 0.05% NaClO added, in 9-cm Petri dishes as described by Seifert et al. (1993). Hyphal tips of emerging colonies were cut and transferred to MEA plates in order to obtain pure strains. The strains were grown routinely on 2% MEA at 25 °C. Representative cultures of each morphotype were deposited in the China Forestry Culture Collection Center (CFCC, part of the National Infrastructure of Microbial Resources) and the culture collection of the Chinese Academy of Forestry (CXY) (Table 2).

**Table 2.** Representative strains of the ophiostomatoid fungi associated with three *Tomicus* spp. in Yunnan Province, China, and three *E.vermicola* strains used in this study.

**Group**	**Taxon**	**Strain no.**	**Host**	**Location**	**Beetle**	**GenBank no.**				
						LSU	ITS\ ITS2–LSU5	BT	EF	CAL
A	* Esteya vermicola *	CFCC52625 (CXY1893)	* P. yunnanensis *	Xiangyun	* T. yunnanensis *	MH325143	–	MH697597	MH605999	–
		ATCC74485	Japanese black pine	Taiwan, China	* Bursaphelenchus xylophilus *	–	–	–	GQ995674	–
		CNU120806	soil	Korea	saprophytic nematodes	EU627684	–	FJ490553	GQ995671	–
		CBS 115803	oak	Czech Republic	* Scolytus intricatus *	–	–	FJ490552	GQ995672	–
B	*** Graphilbum anningense ***	CFCC52631 (CXY1939)	* P. yunnanensis *	Anning	* T. yunnanensis *	MH325162	MH555903	MH683595	–	–
		CFCC52632 (CXY1940)	* P. yunnanensis *	Anning	* T. yunnanensis *	MH325164	MH555901	MH683596	–	–
		CFCC52633 (CXY1944)	* P. yunnanensis *	Anning	* T. minor *	MH325163	MH555902	MH683597	–	–
C	* Leptographium yunnanense *	CFCC52619 (CXY1897)	* P. kesiya *	Ninger	* T. brevipilosus *	MH325138	MH487721	MH603933	MH606000	–
		CFCC52620 (CXY1900)	* P. yunnanensis *	Xiangyun	* T. yunnanensis *	MH325139	MH487724	MH603934	MH606001	–
		CFCC52621 (CXY1904)	* P. yunnanensis *	Xiangyun	* T. yunnanensis *	MH325140	MH487726	MH603935	MH606003	–
		CFCC52622 (CXY1908)	* P. yunnanensis *	Xiangyun	* T. yunnanensis *	MH325142	MH487725	MH603938	MH606002	–
		CFCC52623 (CXY1917)	* P. kesiya *	Puer	* T. brevipilosus *	MH325137	MH487723	MH603936	MH606004	–
		CFCC52624 (CXY1925)	* P. yunnanensis *	Xiangyun	* T. yunnanensis *	MH325141	MH487722	MH603937	MH606005	–
D	* Ophiostoma brevipilosi *	CFCC52596 (CXY1828)	* Pinus kesiya *	Puer	* T. brevipilosus *	MH325134	MH555904	MH619527	–	–
		(CXY1806) CFCC52597	* P. kesiya *	Puer	* T. brevipilosus *	MH325135	MH555905	MH619528	–	–
		CFCC52598 (CXY1808)	* P. kesiya *	Puer	* T. brevipilosus *	MH325136	MH555906	MH619529	–	–
E	* O. canum *	CFCC52601 (CXY1858)	* P. yunnanensis *	Xiangyun	* T. minor *	MH325151	MH555889	MH619521	–	–
		CFCC52602 (CXY1848)	* P. yunnanensis *	Xiangyun	* T. minor *	MH325152	MH555890	MH619522	–	–
		CFCC52603 (CXY1857)	* P. yunnanensis *	Xiangyun	* T. minor *	MH325153	MH555891	MH619523	–	–
F	*** O. aggregatum ***	CFCC52615 (CXY1876)	* P. yunnanensis *	Xiangyun	* T. yunnanensis *	MH325146	MH555894	MH603927	–	–
		CFCC52616 (CXY1875)	* P. yunnanensis *	Xiangyun	* T. yunnanensis *	MH325145	MH555893	MH603929	–	–
		CFCC52617 (CXY1874)	* P. kesiya *	Puer	* T. minor *	MH325147	MH555895	MH603928	–	–
G	* O. minus *	CFCC52606 (CXY1885)	* P. yunnanensis *	Xiangyun	* T. yunnanensis *	MH325154	MH578163	MH619524	–	–
		CFCC52607 (CXY1877)	* P. yunnanensis *	Xiangyun	* T. yunnanensis *	MH325155	MH578164	MH619525	–	–
		CFCC52608 (CXY1881)	* P. yunnanensis *	Xiangyun	* T. yunnanensis *	MH325156	MH578165	MH619526	–	–
H	*O.tingen*s	CFCC52611 (CXY1866)	* P. yunnanensis *	Xiangyun	* T. minor *	MH325148	MH578166	MH603931	–	–
		CFCC52612 (CXY1865)	* P. yunnanensis *	Xiangyun	* T. minor *	MH325149	MH578167	MH603932	–	–
		CFCC52613 (CXY1868)	* P. yunnanensis *	Xiangyun	* T. yunnanensis *	MH325150	MH578168	MH603930	–	–
I	*Ophiostoma* sp. 1	CFCC52618 (CXY1936)	* P. yunnanensis *	Xiangyun	* T. yunnanensis *	MH325144	MH555892	MH683600	–	–
J	*** Sporothrix macroconidia ***	CFCC52628 (CXY1894)	* P. yunnanensis *	Xiangyun	* T. yunnanensis *	MH325157	MH555898	MH697594	–	MH592598
		CFCC52629 (CXY1895)	* P. kesiya *	Ninger	* T. brevipilosus *	MH325158	MH555899	MH697595	–	MH592599
		CFCC52630 (CXY1896)	* P. kesiya *	Ninger	* T. brevipilosus *	MH325159	MH555900	MH697596	–	MH592600
K	*** S. pseudoabietina ***	CFCC52626 (CXY1937)	* P. yunnanensis *	Qujing	* T. minor *	MH325160	MH555896	MH683598	–	MH592601
		CFCC52627 (CXY1938)	* P. yunnanensis *	Qujing	* T. minor *	MH325161	MH555897	MH683599	–	MH592602

Species names in bold are species newly described in this study. CFCC: China Forestry Culture Collection Center, Beijing, China; CXY (Culture Xingyao): Culture collection of the Research Institute of Forest Ecology, Environment and Protection, Chinese Academy of Forestry. Sequences missing data are indicated by [–]

### Morphology and growth studies

Morphological characterisation of both the sexual and asexual reproduction forms was performed on 2% MEA media incubated 3–6 weeks at 25 °C in the dark. Slide cultures were made to observe all microscopic characters (sexual/asexual structures) using a BX51 OLYMPUS microscope with differential interference contrast. Fifty measurements were made of each relevant structure and the ranges were calculated. Standard deviation (*SD*), minimum (min) and maximum (max) measurements are presented as (min–) (mean–*SD*) – (mean+*SD*) (–max).

The optimal growth temperature of the various strains was determined by placing a 5-mm (diam.) plug from an actively growing fungal colony upside down at the centre of an MEA plate. For each strain, three replicates were incubated at temperatures ranging from 5 to 35 °C at five-degree intervals, for 8d. The diameter of each colony was measured daily. Culture characters were recorded on MEA incubated at 25 °C for 8 d and 20 d. Colour descriptions were made by reference to Rayner (1970).

### DNA extraction and sequencing

DNA was extracted from actively growing mycelium scraped from seven-day-old cultures using sterile scalpels and transferred to 2 ml Eppendorf tubes. DNA extraction and purification were performed using the Invisorb Spin Plant Mini Kit (Invitek, Berlin, Germany), following the manufacturer’s protocols.

DNA sequences were determined for six gene regions: the nuclear ribosomal large subunit region (LSU), the internal transcribed spacer regions 1 and 2, including the intervening 5.8S gene (ITS), as well as segments of the β-tubulin (*TUB2*), elongation factor 1α (*TEF1-α*) and calmodulin (*CAL*) genes. DNA fragments were amplified using the primer pairs LROR/LR5 (Vilgalys and Hester 1990), ITS1/ITS4 (White et al. 1990), ITS3/LR3 (Vilgalys and Hester 1990, White et al. 1990), Bt2a/Bt2b (Glass and Donaldson 1995), EF1/EF2 (Jacobs et al. 2004) and CL1/CL2a (Zhang et al. 2015), respectively. PCR reactions were conducted in 25 μl volumes (2.5 mM MgCl_2_, 1× PCR buffer, 0.2 mM dNTP, 0.2 mM of each primer and 2.5 U Taq-polymerase enzyme). PCR amplifications were carried out in a thermocycler (Applied Biosystems, Foster City, California, USA). The reaction conditions for these six gene regions were similar to those described in the references used for primer design. PCR products were cleaned with an MSB Spin PCR apace Kit (250), following the manufacturer’s instructions.

### Phylogenetic analyses

BLAST searches for the obtained sequences were performed in NCBI GenBank and published sequences of closely related species were downloaded. Alignments of the genes were made using MAFFT 7.0 (Katoh and Standley 2013) and the E-INS-i strategy and edited manually in MEGA 5.2 (Tamura et al. 2011). Phylogenetic analyses were performed using maximum parsimony (MP) as well as maximum likelihood (ML).

ML analyses were implemented using RAxML v. 7.0.3 (Stamatakis 2006), under the GTR-GAMMA model. Support for the nodes was estimated from 1 000 bootstrap replicates. The results were subsequently exported to Figtree v.1.4.2 to visualise the trees.

MP analyses were implemented in PAUP* 4.0b10 (Swofford 2003). The most parsimonious trees were identified by a heuristic search of 1 000 random addition sequence replicates, using the tree-bisection-recognition (TBR) algorithm for branch swapping. Branch support was assessed by 1 000 bootstrap replicates. Tree length (TL), consistency index (CI), retention index (RI), rescaled consistency index (RC) and homoplasy index (HI) were used to evaluate the trees.

## Results

### Fungal isolation and sequence comparisons

Three *Tomicus* species occurred on *P.yunnanensis* and *P.kesiya* in the areas studied, either independently or concomitantly in individuals of the host trees (Fig. 1). In total, 772 strains of ophiostomatoid fungi (*Hyalorhinocladiella*-like, *Ophiostoma*, *Pesotum*-like, *Leptographium*-like and *Sporothrix*-like) were isolated from 223 adult beetles (20% of the strains) and 890 galleries (80% of the strains). Galleries or adults of *T.yunnanensis* yielded 297 strains whereas 247 strains were retrieved from galleries or adults of *T.minor* and 228 strains from galleries or adults of *T.brevipilosus* (Table 3).

The LSU sequence was used to search for preliminary affinities using the BLASTn search option in GenBank. As a result, these strains were found to be distributed over 5 genera and 11 tentative species/groups (A–K) (Table 2).

### Phylogenetic analyses

**Figure 2.**
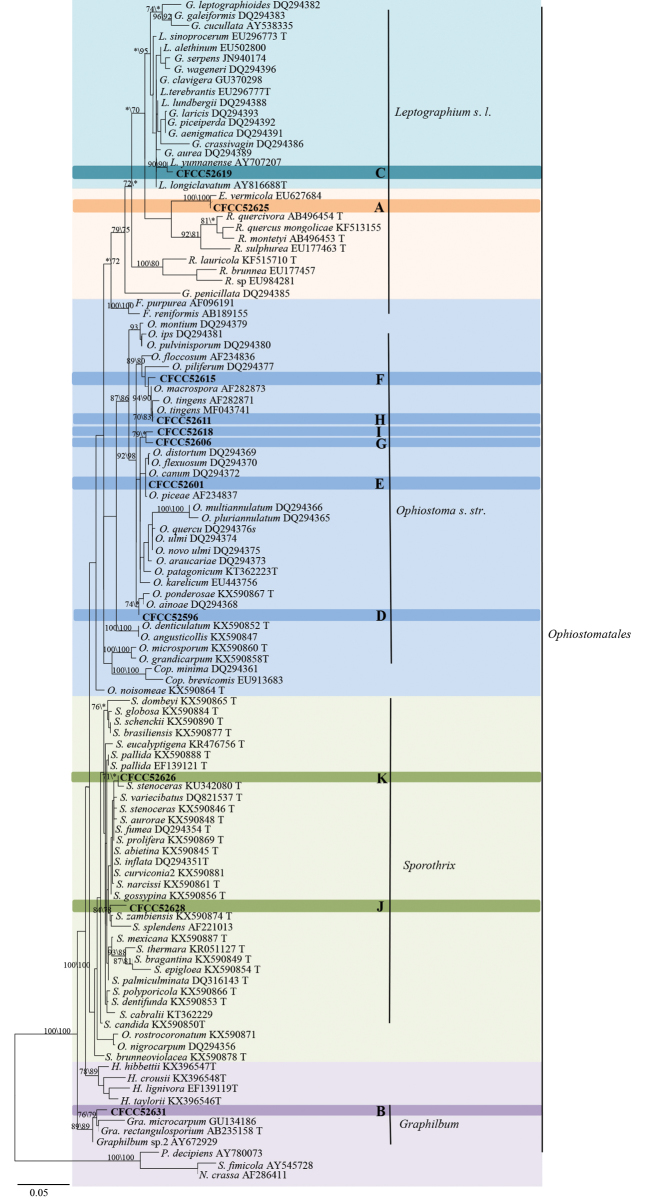
Phylograms obtained from ML analysis of LSU sequences, showing fungal associates with pines infected by *Tomicusyunnanensis*, *T.minor* and *T.brevipilosus* in Yunnan Province, China. Novel sequences obtained in this study are printed in bold type. Bootstrap values ≥ 70% for ML and MP are indicated above branches. Bootstrap values < 70% are indicated by the symbol *. Strains representing ex-type sequences are marked with ‘T’; ML, maximum likelihood; MP, maximum parsimony and the final alignment of 743 positions, including gaps.

The degrees of polymorphism of LSU, ITS, *TUB2*, *TEF1-α* and *CAL* make them variably suitable for genus or species discrimination amongst ophiostomatoid fungi. The LSU sequence is a suitable marker to infer the generic affinities (de Beer and Wingfield 2013, de Beer et al. 2013a, 2016); it allowed confirming the preliminary placement of our strains based on morphological characters (Fig. 2). The ITS region would be useful to place strains within the *Ophiostomas. l.* complex, but the degree of polymorphism does not allow distinguishing species. Usually, *TUB2*, *TEF1-α* and *CAL* regions are better markers to identify and, where appropriate, to show the genetic diversity within ophiostomatoid fungi (Zipfel et al. 2006, de Beer and Wingfield 2013, de Beer et al. 2016).

On the basis of the LSU blast searches, one to six strains of each tentative species (A–K) were selected for sequencing of five additional DNA markers (ITS, ITS2-LSU, *TUB2*, *TEF1-α* and *CAL*) to infer more accurate identification and phylogenetic affinities. Six sequence datasets (LSU, ITS, ITS2-LSU, *TUB2*, *TEF1-α* and *CAL*) were generated for a total of 31 representative strains (Table 2) and the sequences were deposited in GenBank. Resulting alignments were deposited in TreeBASE (submission no: 24032). The topologies generated by the ML and MP analyses were highly concordant and the ML phylograms are presented for all the individual genes, incorporating nodal supports of both the ML and MP analyses.

The LSU dataset consisted of 109 sequences, 11 sequences obtained in this study and 98 downloaded from GenBank. The phylogenetic analyses confirmed the morphology-based placement of our strains into *Esteya*, *Graphilbum*, *Leptographium*, *Ophiostoma* and *Sporothrix* (Fig. 2).

**Figure 3.**
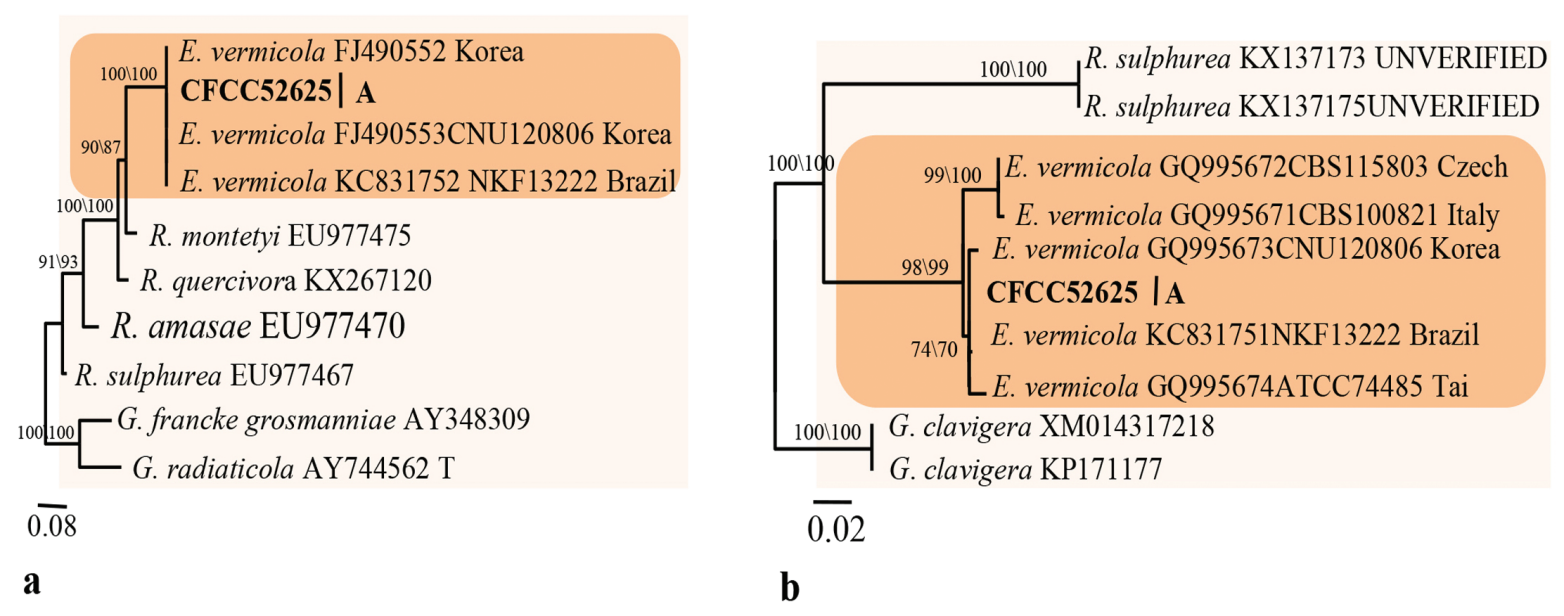
Phylograms obtained from ML analysis of β-tubulin **A** and elongation factor **B** sequences of *Esteya*, showing fungal associates with pines infected by *Tomicusyunnanensis* in Yunnan Province, China. Novel sequences obtained in this study are printed in bold type. Bootstrap values ≥ 70% for ML and MP are indicated above branches. Bootstrap values < 70% are indicated by the symbol *. Strains representing ex-type sequences are marked with ‘T’; ML, maximum likelihood; MP, maximum parsimony and the final alignment of 320 (**A**), 856 (**B**) positions, including gaps.

Group A consisted of a single strain. LSU-based phylogenetic analysis showed this strain to be close to *E.vermicola* (Fig. 2). *TUB2* and *TEF1-α* data analysis confirmed the strain’s close affinities to *E.vermicola* (Fig. 3a, b), that could justify conspecificity.

**Figure 4.**
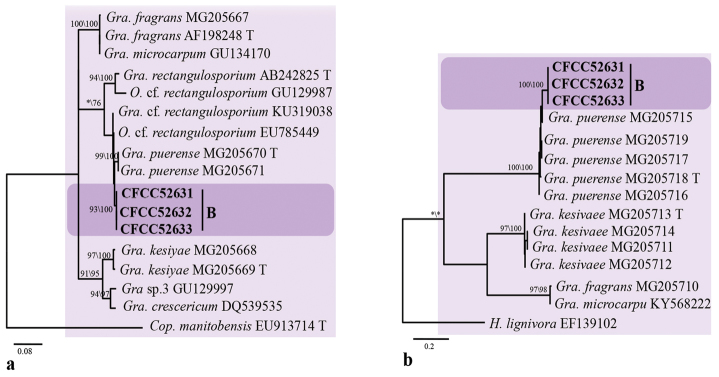
Phylograms obtained from ML analysis of ITS sequences **A** and β-tubulin sequences **B** of *Graphilbum* showing fungal associates with pines infected by *Tomicusyunnanensis* and *T.minor* in Yunnan Province, China. Novel sequences obtained in this study are printed in bold type. Bootstrap values ≥ 70% for ML and MP are indicated above branches. Bootstrap values < 70% are indicated by the symbol *. Strains representing ex-type sequences are marked with ‘T’; ML, maximum likelihood; MP, maximum parsimony and the final alignment of 515 (**A**), 481 (**B**) positions, including gaps.

Group B strains nested within the *Graphilbum* lineage in the LSU-based phylogenetic analysis (Fig. 2). Phylogenetic analysis based on LSU, ITS and *TUB2* concordantly showed that the group B strains formed a single, well-supported clade related to but distinct from *Gra.rectangulosporium* and *Gra.microcarpum* (Fig. 4a, b); this would warrant its recognition as a distinct, undescribed species.

**Figure 5.**
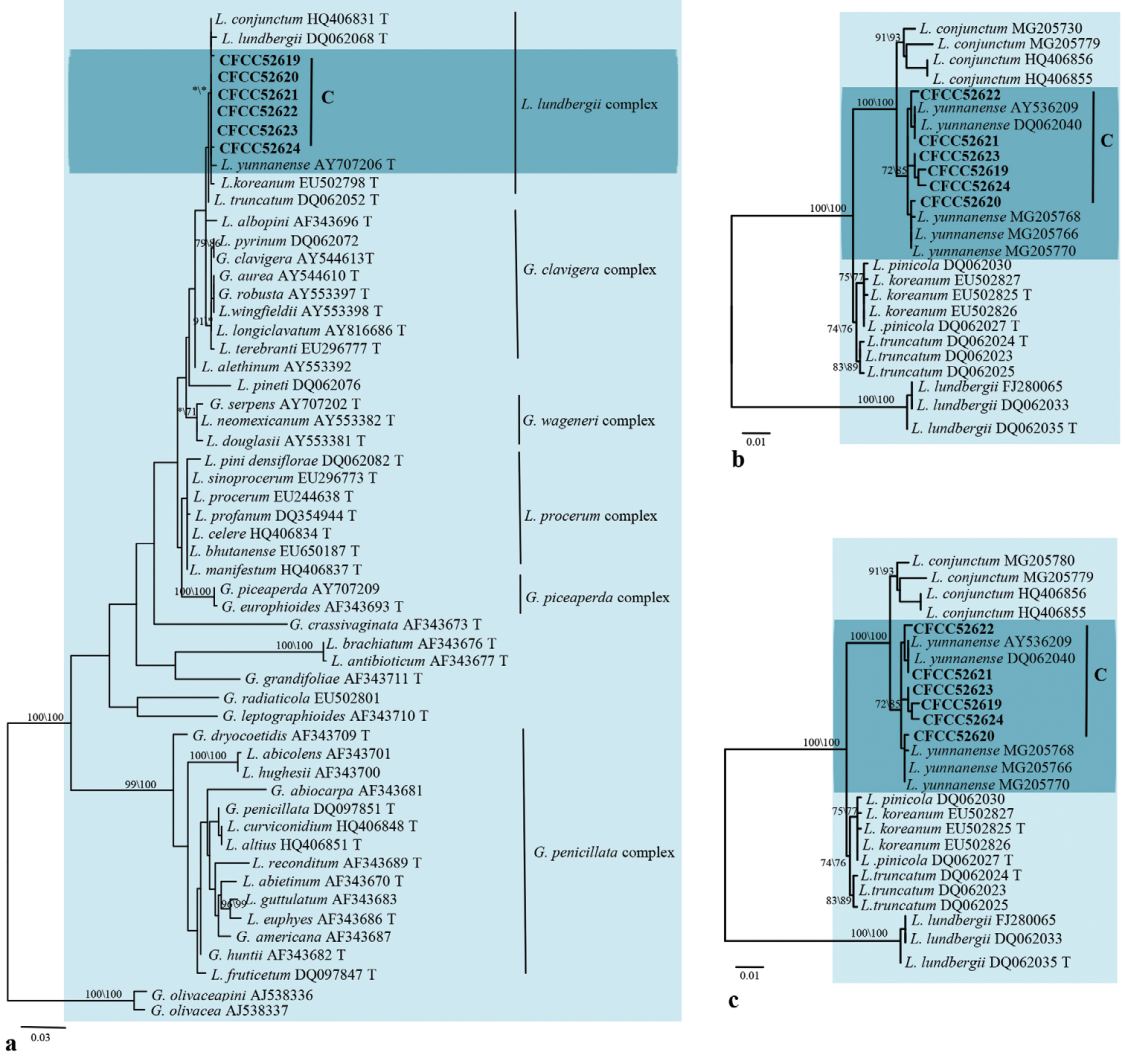
Phylograms obtained from ML analysis of ITS2-28S **A** β-tubulin **B** and elongation factor **C** sequences of *Leptographium*, showing fungal associates with pines infected by *Tomicusyunnanensis* and *T.brevipilosus* in Yunnan Province, China. Novel sequences obtained in this study are printed in bold type. Bootstrap values ≥ 70% for ML and MP are indicated above branches. Bootstrap values < 70% are indicated by the symbol *. Strains representing ex-type sequences are marked with ‘T’; ML, maximum likelihood; MP, maximum parsimony and the final alignment of 641 (**A**), 358 (**B**), 639 (**C**) positions, including gaps.

Group C strains were shown to belong to the *Leptographium* lineage in the LSU-based phylogenetic analysis (Fig. 2). The ITS2-LSU dataset consisted of six of our own sequences and 49 reference sequences downloaded from GenBank. Within the *Leptographium* lineage, group C strains nested in the *L.lundbergii*-complex; they were related to *L.yunnanense*, *L.lundbergii* and *L.conjunctum* (Fig. 5a). *TUB2*- and *TEF1-α* based analysis confirmed their close affinities with *L.yunnanense*, although forming a slightly divergent clade (Fig. 5b, c). *TUB2* and *TEF1-α* sequences of group C strains showed some polymorphisms, which could be considered as falling within the natural diversity of *L.yunnanense*.

**Figure 6.**
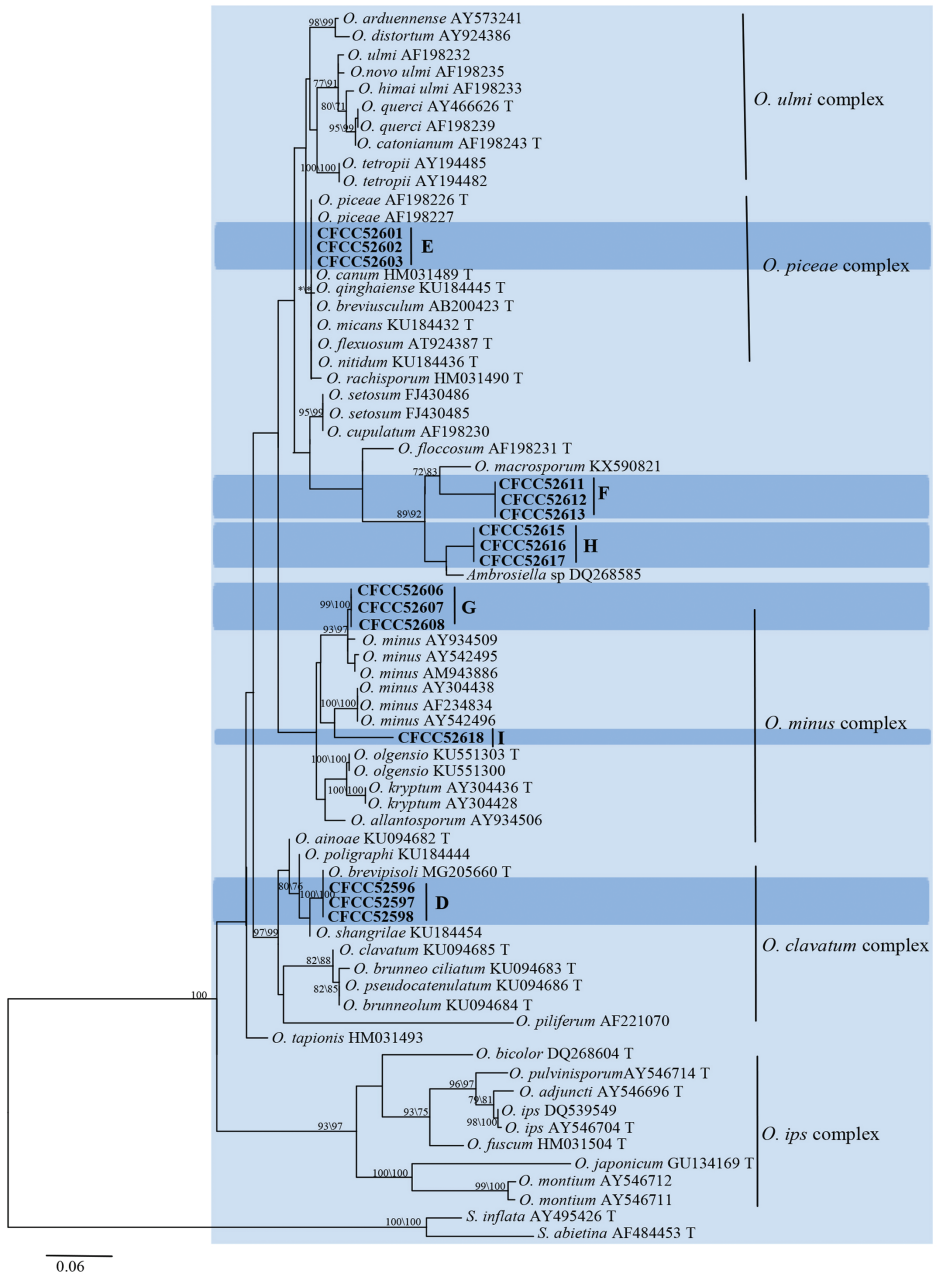
Phylograms obtained from ML analysis of ITS sequences of *Ophiostoma*, showing fungal associates with pines infected by *Tomicusyunnanensis*, *T.minor* and *T.brevipilosus* in Yunnan Province, China. Novel sequences obtained in this study are printed in bold type. Bootstrap values ≥ 70% for ML and MP are indicated above branches. Bootstrap values < 70% are indicated by the symbol *. Strains representing ex-type sequences are marked with ‘T’; ML, maximum likelihood; MP, maximum parsimony and the final alignment of 633 positions, including gaps.

The six strains from groups D to I nested within the *Ophiostoma* lineage based on the LSU phylogenetic tree (Fig. 2). The ITS dataset comprised species from all lineages discovered in this study. Analysis of this dataset yielded the phylograms shown in Fig. 6. Sixteen ITS sequences generated in this study were compared with 61 sequences retrieved from GenBank, representing the major groups of *Ophiostoma* (de Beer and Wingfield 2013, Linnakoski et al. 2016).

**Figure 7.**
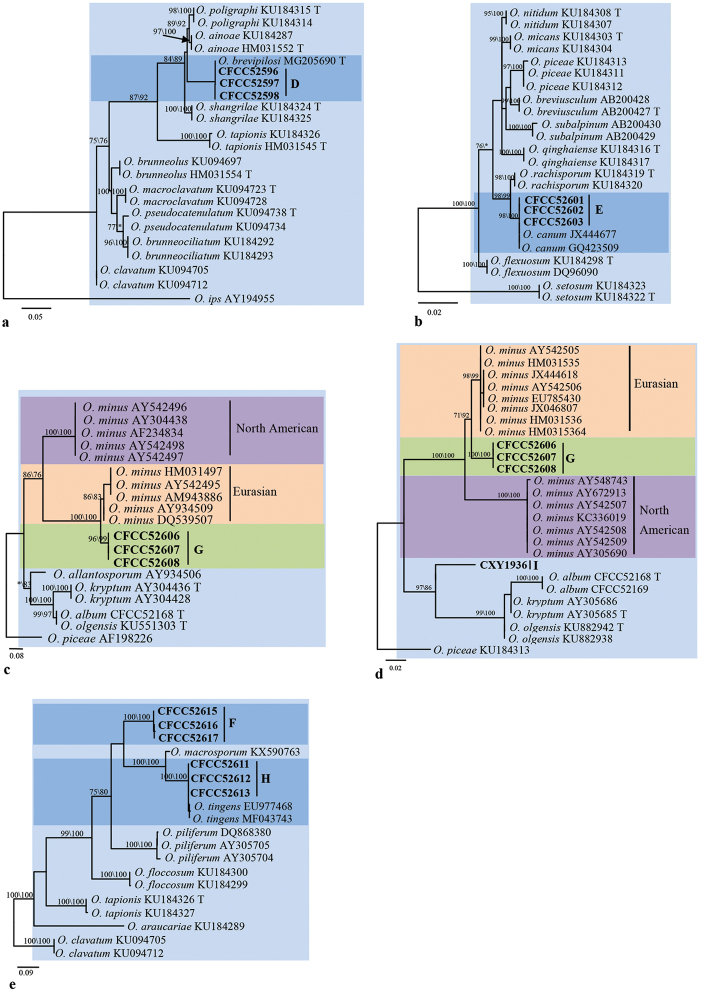
Phylograms obtained from ML analysis of β-tubulin sequences of *Ophiostoma***A, B, D, E** and ITS sequences of *O.minus*-complex **C** showing fungal associates with pines infected by *Tomicusyunnanensis*, *T.minor* and *T.brevipilosus* in Yunnan Province, China. Novel sequences obtained in this study are printed in bold type. Bootstrap values ≥ 70% for ML and MP are indicated above branches. Bootstrap values < 70% are indicated by the symbol *. Strains representing ex-type sequences are marked with ‘T’; ML, maximum likelihood; MP, maximum parsimony and the final alignment of 455 (**A**), 430 (**B**), 541 (**C**), 378 (**D**), 423 (**E**) positions, including gaps.

The ITS- and *TUB2*-based phylogenetic inferences (Figs 6, 7a, b) showed that the strains of groups D and E nested within the *O.clavatum*- and *O.piceae*-complex (de Beer and Wingfield 2013, Yin et al. 2016, Linnakoski et al. 2016), in which they were positioned in the near vicinity of the *O.brevipilosi* and *O.canum* clades, respectively. From these results, and considering their morphological features, we concluded that the strains of groups D and E are conspecific with *O.brevipilosi* and *O.canum*, respectively.

In the ITS-based phylogenetic analysis, strains of groups G and I were grouped with the *O.minus* complex (Fig. 6). ITS- and *TUB2*-based phylogenetic analyses consistently showed that group G strains formed a well-supported subclade between the North American and European subclades within the *O.minus* lineage (Fig. 7c, d). The strains of group G are therefore identified as *O.minus*. The ITS- and *TUB2*-based phylogenetic analyses consistently showed that the single strain of group I formed a branch that is related to, but distinct from the *O.minus*, *O.kryptum* and *O.olgensis* clades (Figs 6, 7d). Hence, this strain is interpreted as belonging to a distinct, undescribed *Ophiostoma*.

The remaining two groups (F and H) were not placed in any defined complex. Phylogenetic analyses, based on ITS and *TUB2* sequences, consistently showed that the group H strains clustered in the near vicinity of the *O.tingens* clade whereas group F strains formed a clade related to, but distinct from the *O.macrosporum* and *O.tingens* clades (Figs 6, 7e). Thus, the strains in group H should be identified as *O.tingens* whereas the strains of group F represent an undescribed *Ophiostoma*.

**Figure 8.**
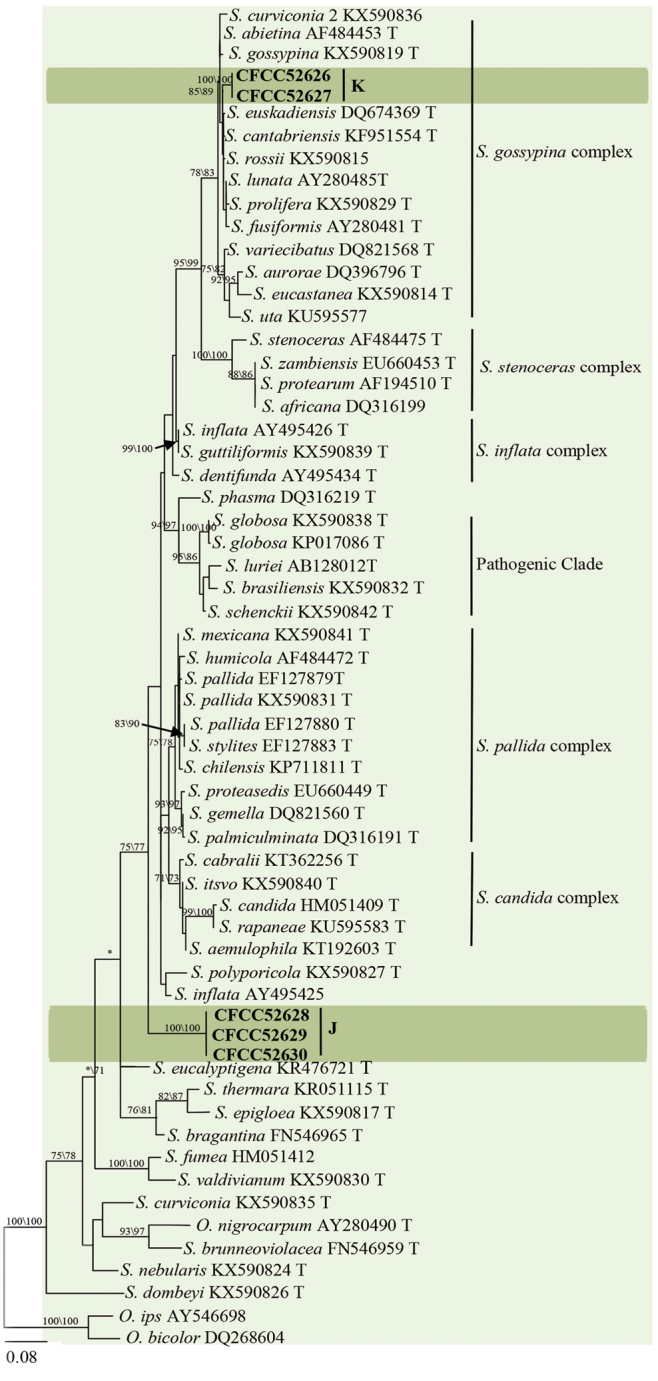
Phylograms obtained from ML analysis of ITS sequences of *Sporothrix*, showing fungal associates with pines infected by *Tomicusyunnanensis*, *T.minor* and *T.brevipilosus* in Yunnan Province, China. Novel sequences obtained in this study are printed in bold type. Bootstrap values ≥ 70% for ML and MP are indicated above branches. Bootstrap values < 70% are indicated by the symbol *. Strains representing ex-type sequences are marked with ‘T’; ML, maximum likelihood; MP, maximum parsimony and the final alignment of 546 positions, including gaps.

**Figure 9.**
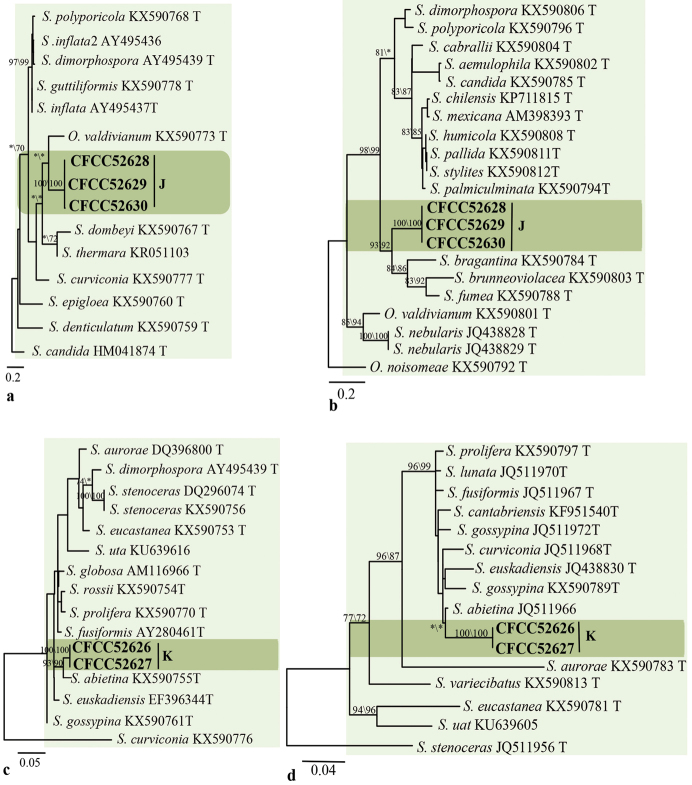
Phylograms obtained from ML analysis of β-tubulin **A, C** and calmodulin **B, D** sequences of *Sporothrix*, showing fungal associates with pines infected by *Tomicusyunnanensis*, *T.minor* and *T.brevipilosus* in Yunnan Province, China. Novel sequences obtained in this study are printed in bold type. Bootstrap values ≥ 70% for ML and MP are indicated above branches. Bootstrap values < 70% are indicated by the symbol *. Strains representing ex-type sequences are marked with ‘T’; ML, maximum likelihood; MP, maximum parsimony and the final alignment of 284(**A**), 622(**B**), 260(**C**), 675(**D**) positions, including gaps.

Strains of groups J and K nested within the *Sporothrix* lineage in LSU-based phylogenetic analysis (Fig. 2). The phylograms resulting from the analyses of individuals are shown in Fig. 8 (ITS), Fig. 9a, c (*TUB2*) and Fig. 9b, d (CAL).

The ITS-based analyses showed that group K strains belonged to the *S.gossypina*-complex whereas the group J strains were not placed in any species complex as defined by de Beer et al. (2016) (Fig. 8). Both groups formed independent, well-supported clades in ITS-, *TUB2*- and *CAL*-based phylogenetic analyses (Figs 8, 9). It could be deduced from results of multiple phylogenies that both groups represent novel species.

## Morphology and taxonomy

From a morphological perspective, strains of groups D, E and G appeared, overall, concordant with the descriptions or our own observations of reference strains, namely of *O.brevipilosi*, *O.canum* and *O.minus*, respectively. However, although strains of groups A, C, and H are phylogenetically close to *E.vermicola*, *L.yunnanense* and *O.tingens*, respectively, justifying, for the time being, conspecificity, their phenotype deviated slightly from published descriptions and/or our own observation of type material. The description of these species is extended. Strains of groups B, F, J and K revealed unique combinations of phenotypes, allowing morphological distinction from their closest phylogenetic relatives; consequently, they are described below as new species. The strain of the stand-alone group I also may represent an undescribed species; however, we refrain from describing it for the time being, waiting for more material to become available.

### Taxonomy

#### Esteya vermicola

Taxon classificationFungiOphiostomatalesOphiostomataceae

J.Y. Liou, J.Y. Shih & Tzean, Mycol. Res. 103(2): 243. 1999.

MB450702

Fig. 10

##### Description.

Sexual form: unknown.

Asexual form: *Hyalorhinocladiella*-like. *Conidiophores* mononematous, micronematous; *conidiophorous cells* solitary, integrated, flask-shaped, with an inflated base (3.6–) 4.6–6.1 (–7.1) μm in diam., the fertile hyphoid part (9.1–) 12.2–19.0 (–22.5) × (1.4–) 1.9–3.1 (–4.7) μm, often crooked due to successive conidial development; *conidia* 1-celled, asymmetrically ellipsoidal in face view, concave, lunate in side view, with a layer of adhesive mucus on the concave surface, ending slightly apiculate, hyaline, smooth, (8.0–) 10–12 (–13.1) × (3.3–) 3.4–4.5 (–5.1) μm, containing an ovoid endospore-like structure.

**Figure 10.**
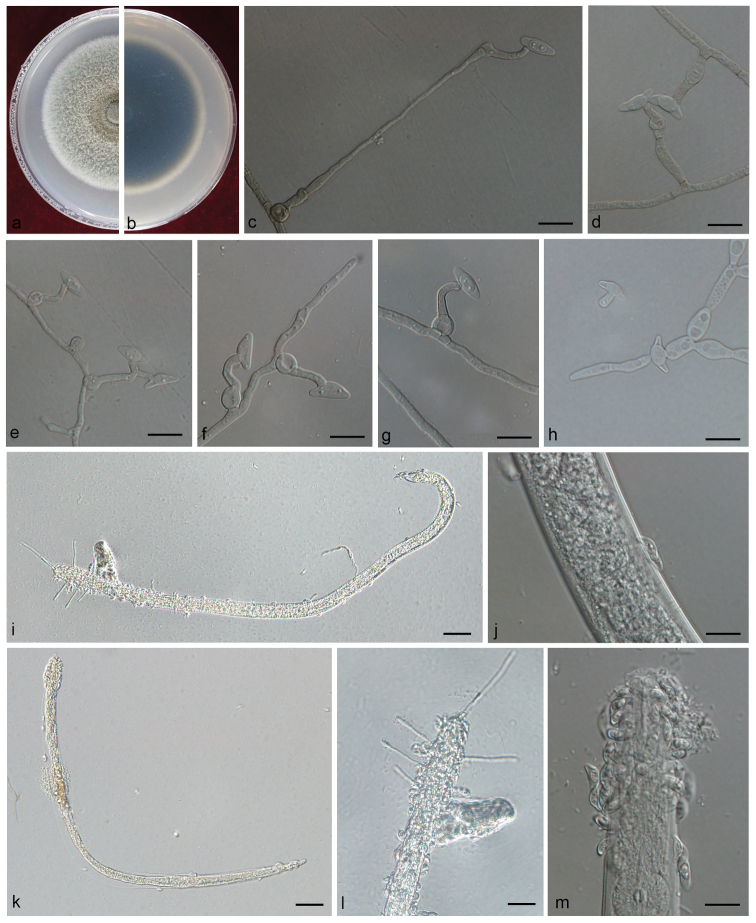
**A–H** Morphological characters of *Esteyavermicola***A, B** upper and reverse of cultures on 2% MEA 20 d after inoculation **C–H** conidiogenous cells with lunate conidia **I–M** the cuticle of a nematode attached by many lunate conidia. Scale bars: 20 μm (**I, K, L)**; 10 μm (**C–H, J, M**).

##### Culture characteristics.

Colonies on 2% MEA in the dark reaching 31 mm in diam. in 8 days at 25 °C, growth rate up to 5 mm/day at the fastest; colony margin smooth. Mycelium compact, somewhat floccose in the margin, white at first, gradually discolouring to greyish-green, eventually dark green. Optimal growth temperature 25 °C, growth at 5 °C and 35 °C.

##### Known substrate and host.

Galleries of *Tomicusyunnanensis* in *Pinusyunnanensis*.

##### Known insect vector.

*Tomicusyunnanensis*.

##### Known distribution.

Yunnan Province, China.

##### Specimen examined.

CHINA, Yunnan, *Tomicusyunnanensis* galleries in *Pinusyunnanensis*, Dec. 2016, HM Wang, CFCC 52625 = CXY 1893.

##### Note.

*Esteyavermicola* is known only from an asexual, *Hyalorhinocladiella*-like state producing lunate and bacilliform conidia (Liou et al. 1999, Kubátová et al. 2000, Wang et al. 2009, 2014) that we also observed in various strains of *E.vermicola* with a different origin (Taiwan, Korea, Czech Republic). Our strain was identified as *E.vermicola* based on phylogenetic inferences and morphological characters. However, our strain differed from previous descriptions (Liou et al. 1999) in having only lunate conidia *in vitro*. The size of the lunate conidia of our strains (mostly 10 - 12 × 3.4 - 4.5 μm) was similar to that reported for *E.vermicola*, *viz.* 9.9–11.9 × 3.4–4.5 μm vs 8.2-11.1 × 3.5–3.7 μm (Taiwan, Liou et al. 1999), 9.3–12.4 × 3.0–3.2 μm (Czech Republic, Kubátová et al. 2000), 7.7–12.1 × 3.0–3.8 μm (Korea, Wang et al. 2009) or 8.7–11.9 × 3.0–3.6 μm (Brazil, Wang et al. 2014).

This is the first report of *E.vermicola* from continental China. The species was originally isolated from Japanese black pine infected by the pinewood nematode *Bursaphelenchusxylophilus*, in Taiwan (Liou et al. 1999). Since then, its distribution range has been extended to Japan and Korea, Europe (Czech Republic, Italy) and both North (USA) and South America (Brazil) (Liou et al. 1999, Kubátová et al. 2000, Wang et al. 2009, 2014, Li et al. 2018). This species is associated with various vectors, including the pinewood nematode, *Oxoplatypusquadridentatus* and the bark beetle *Scolytusintricatus*. It was isolated also from wooden packaging material infested by *Bursaphelenchusrainulfi*.

#### Graphilbum anningense

Taxon classificationFungiOphiostomatalesOphiostomataceae

H. Wang, Q. Lu & Z. Zhang sp. n.

MB828884

Fig. 11

##### Etymology.

‘*anningense*’ (Latin), referring to the type locality.

##### Type.

**CHINA**, Yunnan, *Tomicusyunnanensis* galleries in *Pinusyunnanensis*, Apr. 2017, HM Wang, holotype CXY 1939, culture ex-holotype CFCC 52631 = CXY 1939.

##### Description.

Sexual form: unknown.

Asexual forms: *Pesotum*-like and *Hyalorhinocladiella*-like. *Pesotum*-like *conidiophores* abundant on 2% MEA, macronematous, synnematous, (150–) 210–293 (–336) μm long including conidiogenous apparatus, the base dark brown, slightly widened, (6.7–) 7.9–18.8 (–29.0) μm wide anchored in the media by brown rhizoid-like hyphae, the apex slightly enlarging, fan-shaped; *conidiogenous cells* hyaline, thin-walled, aseptate, (15.3–) 21.0–35.5 (–42) × (0.7–) 1.1–1.9 (–2.3) μm; *conidia* 1-celled, clavate, ellipsoid to ovoid with truncate base and rounded apex, hyaline, smooth, (3.1–) 3.6–6.3 (–9.7) × (1.4–) 1.6–2.2 (–2.5) μm.

**Figure 11.**
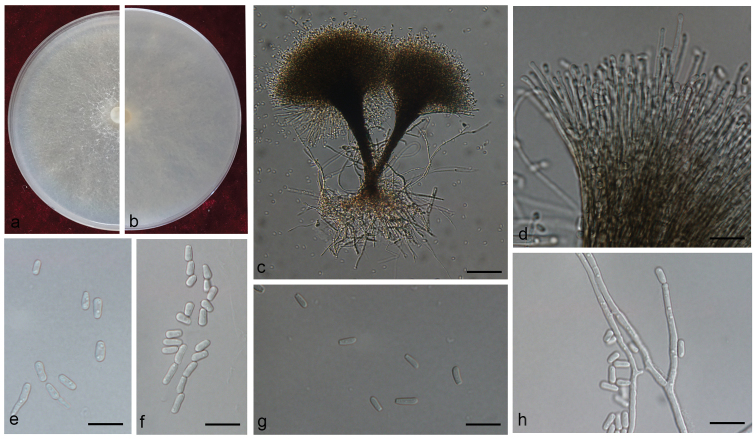
Morphological characters of *Graphilbumanningense* sp. n. **A, B** Upper and reverse of cultures on 2% MEA 8 d after inoculation **C, D, G** conidiogenous cells of Pesotum-like macronematal asexual state and conidia **E, F, H** conidiogenous cells of *Hyalorhinocladiella*-like asexual state and conidia. Scale bars: 50 μm (**C)**; 20 μm (**D)**; 10 μm (**E–H)**.

*Hyalorhinocladiella*-like: *conidiogenous cells* macronematous or semi-macronematous, mononematous, hyaline, simple or loosely branched, thin-walled, aseptate, (4.5–) 10.8–29.0 (–47) × (1.5–) 1.7–2.3 (–2.6) μm; *conidia* hyaline, clavate to ellipsoid, with obtuse ends, 1-celled, aseptate, smooth, (3.7–) 4.5–6.4 (–9.0) × (1.4–) 1.7–2.3 (–2.9) μm.

##### Culture characteristics.

Colonies on 2% MEA in the dark reaching 90 mm in diam. in 6 days at 25 °C, growth rate up to 19.5 mm/day at the fastest; colony margin smooth. Mycelium superficial to flocculose or floccose, hyaline; reverse hyaline to pale yellowish. Optimal growth temperature 30 °C, slow growth at 40 °C, no growth at 5 °C.

##### Known substrate and hosts.

Galleries of *Tomicusyunnanensis* and *T.minor* in *Pinusyunnanensis*.

##### Known insect vectors.

*Tomicusyunnanensis*, *T.minor*.

##### Known distribution.

Yunnan Province, China.

##### Additional specimens examined.

CHINA, Yunnan, *Tomicusyunnanensis*, *T.minor* galleries in *Pinusyunnanensis*, Apr. 2017, HM Wang, CFCC 52632 = CXY 1940, CFCC 52633 = CXY 1944.

##### Note.

*Graphilbumanningense* is characterised by a *Pesotum*-like and a *Hyalorhinocladiella*-like asexual state. It is phylogenetically closely related to *Gra.rectangulosporium*. However, *Gra.rectangulosporium* produced a sexual state *in vitro* (Ohtaka et al. 2006) which has not been observed in *Gra.anningense*. Other morphologically similar species include *Gra.fragrans*, *Gra.crescericum*, *Gra.kesiyae* and *Gra.puerense*. *Graphilbumkesiyae* and *Gra.puerense* also produce a *Pesotum*-like and a *Hyalorhinocladiella*-like asexual state. *Graphilbumanningense* and *Gra.kesiyae* differ by the size of their synnemata, whose length ranges do not overlap, *viz.* 210–293 μm and 112.5–173 μm long (Harrington et al. 2001), respectively. They also differ by their optimal growth temperature, respectively 30°C and 25°C. The synnemata of *Gra.puerense*, 206–357 μm long (Chang et al. 2017), are marginally longer than those of *Gra.anningense*. *Graphilbumfragrans* and *Gra.crescericum* produce only a *Leptographium*-like and/or a *Hyalorhinocladiella*-like asexual state *in vitro* (Harrington et al. 2001, Chang et al. 2017).

*Graphilbumanningense* was isolated from galleries of *T.yunnanensis* and *T.minor* infesting *P.yunnanensis*. Previously, *Gra.fragrans* had been reported from *T.yunnanensis* infesting *P.yunnanensis* and from *Pissodes* spp. infesting *Tsugadumosa* and *P.armandii* in China (Paciura et al. 2010, Zhou et al. 2013). *Graphilbumkesiyae* and *Gra.puerense* were isolated from galleries of *Polygraphusaterrimus*, *Po.szemaoensis* and *Ipsacuminatus* infesting *P.kesiya* (Chang et al. 2017). Although the geographic distribution of these four Graphilbum species overlaps, their hosts and vectors are nevertheless, as far as it is known, different (Chang et al. 2017).

#### Leptographium yunnanense

Taxon classificationFungiOphiostomatalesOphiostomataceae

X.D. Zhou, K. Jacobs, M.J. Wingf. & M. Morelet, Mycoscience 41(6): 576. 2000.

MB 466542

Fig. 12

##### Description.

Sexual form: unknown.

Asexual form: *Leptographium*-like. *Conidiophores* occurring singly or in groups of up to three, arising from the superficial mycelium, erect, macronematous, mononematous, (93.5–) 159–412 (–544) μm long, without rhizoid-like structures; *stipes* simple, cylindrical, not constricted at septa, 1-6-septate, pale olivaceous at the base, (12–) 19.0–128 (–245) × (3.3–) 4.1–6.1 (–7.3) μm; *conidiogenous apparatus* (33.0–) 65.5–119.5 (–168.0) μm long (high), with 2 to 3 series of cylindrical branches; *primary branches* hyaline to pale olivaceous, smooth, cylindrical, 2–3 septate, (11.5–) 18.2–37.7 (–56.0) μm long and (3.0–) 3.7–5.9 (–7.7) μm wide; *secondary branches* hyaline, 0–2 septate, (10.3–) 14.5–30.0 (–50.1) μm long, (2.8–) 3.4–5.5 (–7.3) μm wide; *conidiogenous cells* discrete, 2–3 per branch, cylindrical, (10.2–) 13.2–29.6 (–57.4) × (2.2–) 2.9–3.9 (–4.4) μm; *conidia* 1-celled, oblong to obovoid with truncate bases, hyaline, (5.8–) 7.0–10.4 (–13.0) × (2.9–) 3.6–5.3 (–6.4) μm.

**Figure 12.**
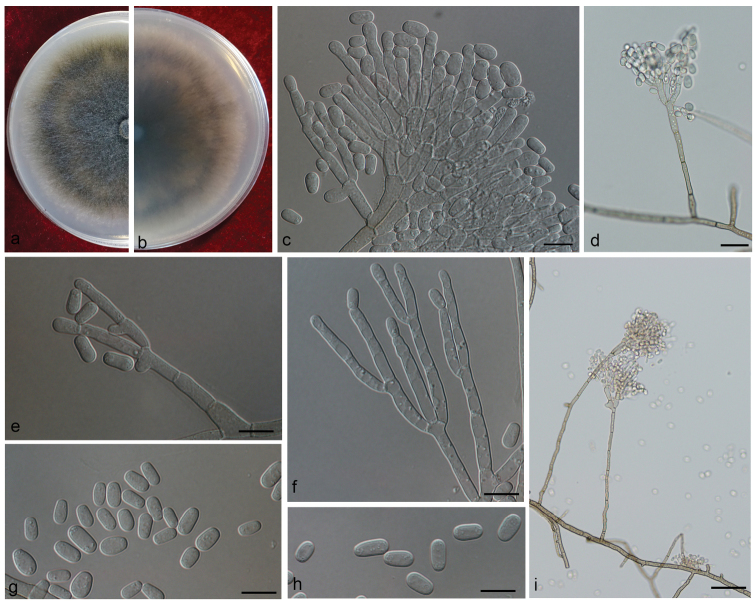
Morphological characters of *Leptographiumyunnanense***A, B** upper and reverse of cultures on 2% MEA 8 d after inoculation **D, I** conidiophore on 2% MEA **C, E–H** conidiogenous cells of *Leptographium*-like asexual state and conidia. Scale bars: 50 μm (**D, I)**; 10 μm (**C, E–H)**.

##### Culture characteristics.

Colonies on 2% MEA medium fast growing in the dark, reaching 76 mm in diam. in 8 days at 25 °C, growth rate up to 20 mm/day at the fastest; colony margin smooth. Hyphae submerged in agar with aerial mycelium, greenish-olivaceous to olivaceous, smooth, straight; reverse hyphae umber-brown to dark olivaceous. Optimal growth temperature 25 °C, slow growth at 5 °C and 30 °C.

##### Known substrate and hosts.

*Tomicusyunnanensis* and its galleries in *Pinusyunnanensis*, galleries of *T.brevipilosus* in *P.kesiya*.

##### Known insect vectors.

*Tomicusbrevipilosus*, *T.yunnanensis*.

##### Known distribution.

Yunnan Province, China.

##### Specimens examined.

CHINA, Yunnan, adults of *Tomicusyunnanensis* and their galleries in *Pinusyunnanensis*, *Tomicusbrevipilosus* galleries in *P.kesiya*. Apr. 2017, HM Wang, CFCC 52619 = CXY 1897, CFCC 52620 = CXY 1900, CFCC 52621 = CXY 1904, CFCC 52622 = CXY 1908, CFCC 52623 = CXY 1917, CFCC 52624 = CXY 1925.

##### Note.

The sole reproductive structure formed on MEA in *L.yunnanense* is a *Leptographium*-like state. Our strains were identified as *L.yunnanense*, based on phylogenetic evidence and secondarily, on morphological features. However, our strains slightly deviated from *L.yunnanense* in having longer conidiophores, mainly 159–412 μm *vs* mostly 74–227 (–233) μm (Zhou et al. 2000) or 80–240 μm (Yamaoka et al. 2008). Furthermore, our strains grew faster than reported for the species, 76 mm *vs* 44 mm in 8 days at 25 °C (Zhou et al. 2000).

Although our strains were slightly genetically and morphologically divergent, we are of the opinion that they enter into the current *L.yunnanense* species concept (e.g. *sensu*Zhou et al. 2000). Yamaoka et al. (2008) showed the genetic diversity of *L.yunnanense* in Yunnan to be higher than in other places, that which is confirmed by the present study.

*Leptographiumyunnanense* was originally described from Yunnan Province with only an asexual state (Zhou et al. 2000). Subsequently, mating of strains from different origins (Thailand, China and Japan) yielded the sexual state, which is formed by neckless ascocarps and cucullate ascospores (Yamaoka et al. 2008).

*Leptographiumyunnanense* was the third most abundant species associated with *T.yunnanensis* in our study. A few strains also were isolated from *T.brevipilosus* infesting *P.kesiya* and none from *T.minor*.

#### Ophiostoma aggregatum

Taxon classificationFungiOphiostomatalesOphiostomataceae

H. Wang, Q. Lu & Z. Zhang sp. n.

MB828885

Fig. 13

##### Etymology.

‘*aggregatum*’ (Latin), reflects to the conidiophores aggregated in clusters.

##### Type.

**CHINA**, Yunnan, from *Tomicusminor* galleries in *Pinusyunnanensis*, Dec. 2016, HM Wang, holotype CXY 1876, culture ex-holotype CFCC 52615 = CXY 1876.

##### Description.

Sexual form: unknown.

Asexual form: *Leptographium*-like. *Conidiophores* macronematous, mononematous, gathered in groups up to 5, (28.5–) 34–45.5 (–52) μm long; *stipes* cylindrical, 1–2 septate, not constricted at septa, umber-brown to dark olivaceous, (6.3–) 7.3–14.5 (–18) μm long × (2.2–) 3.1–4.6 (–5.8) μm wide. *Conidiogenous apparatus* (22–) 26.5–31 (–34) μm long, with 2–3 series of cylindrical branches; primary branches olivaceous, smooth, cylindrical all over, (5.9–) 7.2–13.5 (–20.5) × (3–) 3.3–4.2 (–4.6) μm; *conidiogenous cells* discrete, 2–3 per branch, aseptate, cylindrical, hyaline to pale umber, (5.8) 7.2–12.1 (–18.5) × (2.1–) 2.8–4.0 (–4.7) μm; *conidia* 1-celled, globose, elliptical with truncate bases, hyaline to pale umber, (4.0–) 4.8–5.9 (–6.3) × (3.1–) 4.0–5.0 (–5.6) μm.

**Figure 13.**
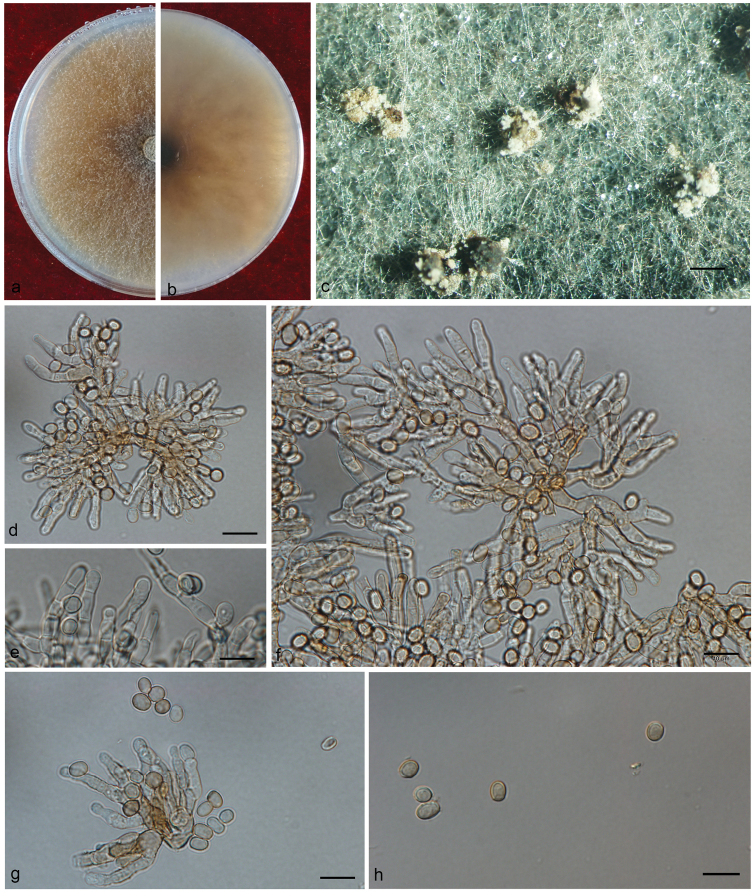
Morphological characters of *Ophiostomaaggregatum* sp. n. **A, B** upper and reverse of cultures on 2% MEA 8 d after inoculation **C** conidiomata on 2% MEA (bar = 50 μm) **D–H** conidiogenous cells of *Leptographium*-like asexual state and conidia. Scale bars: 20 μm (**C)**; 10 μm (**D–H)**.

##### Culture characteristics.

Colonies on 2% MEA fast growing in the dark, reaching 90 mm in diam. in 8 days at 25 °C, growth rate up to 13 mm/day at the fastest; colony margin smooth. Hyphae submerged and aerial, umber-brown to dark olivaceous, flocculose or floccose; reverse hyphae umber-brown to dark olivaceous. Optimal growth temperature 25 °C, able to grow at 5 °C and 30 °C. No growth at 35 °C.

##### Known substrate and hosts.

Galleries of *Tomicusyunnanensis* and *T.minor* in *Pinusyunnanensis*.

##### Known insect vectors.

*Tomicusminor*, *T.yunnanensis*.

##### Known distribution.

Yunnan Province, China.

##### Additional specimens examined.

CHINA, Yunnan, from *Tomicusyunnanensis* and *T.minor* galleries in *Pinusyunnanensis*, Dec. 2016, Apr. 2017, HM Wang, CFCC 52616 = CXY 1875, CFCC 52617 = CXY 1874.

##### Note.

*Ophiostomaaggregatum* produced a single asexual, *Leptographium*-like state *in vitro*. This species is phylogenetically closely related to *O.macrosporum*, *O.tingens*, *O.floccosum*, *O.tapionis* and *O.piliferum* in LSU-, ITS- and *TUB2*-based phylogenetic inferences. *Ophiostomaaggregatum* and *O.tingens* are shown to be sympatric in Yunnan pine forest; both taxa were isolated from galleries and adults of *T.minor* and *T.yunnanensis* infesting *P.yunnanensis* (Table 2). *Ophiostomatingens* was also reported from *T.minor* infesting *P.yunnanensis* in Yunnan (Pan et al. 2017).

*Ophiostomaaggregatum* and *O.tingens* differ in their asexual state. *Ophiostomaaggregatum* only produces a *Leptographium*-like state. Inversely, the asexual states of *O.tingens* are variable. Our strains produced a *Pesotum*-like and a *Sporothrix*-like state whereas previously, Francke-Grosmann (1952) and de Beer et al. (2013b) reported a *Hyalorhinocladiella*- to *Raffaelea*-like state in European strains. The origin of this variability and its importance for taxonomy is uncertain.

*Ophiostomamacrosporum*, *O.floccosum*, *O.tapionis* and *O.piliferum* also differ from *O.aggregatum* by their asexual state. *Ophiostomamacrosporum* and *O.floccosum* produce a *Pesotum*-like asexual state, *O.tapionis* a *Hyalorhinocladiella*-like state and *O.piliferum* produces a *Sporothrix*-like state (Francke-Grosmann 1952, Upadhyay 1981, Yamaoka et al. 2004, Linnakoski et al. 2008).

*Ophiostomamacrosporum* and *O.tingens* were both originally described in *Trichosporium* as T.tingensvar.macrosporum and *T.tingens* (Lagerberg 1927, Francke-Grosmann 1952). Batra (1967) transferred these two species into *Ambrosiella*. It is only recently that the morphological characteristics were found to agree with those of *Ophiostoma* (de Beer et al. 2013b). *Ophiostomamacrosporum* has been reported from various *Pinus* spp. (including *P.sylvestris*) infected by *Ipsacuminatus* in Europe (Francke-Grosmann 1952, Batra 1967).

#### Ophiostoma tingens

Taxon classificationFungiOphiostomatalesOphiostomataceae

(Lagerb. & Melin) Z.W. de Beer & M.J. Wingf., Svensk Skogsvårdsförening Tidskr. 25:233. 1927.

MB801091

Fig. 14

##### Description.

Sexual form: unknown.

Asexual forms: *Pesotum*-like and *Sporothrix*-like. *Pesotum*-like: *conidiophores* macronematous, synnematous; synnemata simple, anchored into the substrate by brown rhizoid-like hyphae, (333–) 344–584 (–684) μm long including *conidiogenous apparatus*, the base dark brown, slightly widened, (16.7–) 17–50.5 (–65.5) μm wide, the apex cream-coloured or pale brown, slightly widening; *conidia* hyaline, globose to elliptical, 1-celled, smooth, (2.7–) 3.6–7.2 (–8.0) × (2.8–) 4.3–6.1 (–7.0) μm.

*Sporothrix*-like: *conidiophores* semi-macronematous, mononematous, hyaline, simple or loosely branched, smooth, bearing terminal denticulate *conidiogenous cells* (8.3–) 15.6–30.0 (–42.5) × (1.1–) 1.7–3.1 (–4.7) μm; *conidia* hyaline, globose to elliptical, obovoid with pointed bases and rounded apices, 1-celled, smooth, (2.6–) 4.0–6.8 (–8.7) × (2.2–) 3.5–5.5 (–7.4) μm.

**Figure 14.**
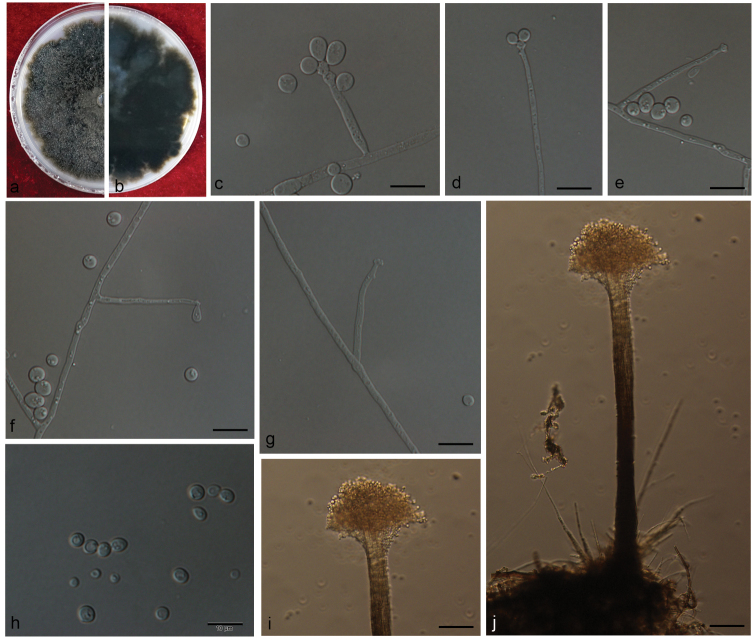
Morphological characters of *Ophiostomatingens***A, B** upper and reverse of cultures on 2% MEA 20 d after inoculation **C–G** conidiogenous cells of *Sporothrix*-like asexual state and conidia **H–J** conidiogenous cells of *Pesotum*-like macronematal asexual state and conidia. Scale bars: 10 μm (**C–H)**; 50 μm (**I, J)**.

##### Culture characteristics.

Colonies on 2% MEA medium slow growing in the dark, reaching 39 mm in diam. in 8 days at 25 °C, growth rate up to 5 mm/day at the fastest; colony margin anomalous. Hyphae appressed to flocculose, black; reverse hyphae also black. Optimal growth temperature 25 °C, no growth at 5 °C and 30 °C.

##### Known substrate and hosts.

Galleries of *Tomicusyunnanensis* and *T.minor* in *Pinusyunnanensis*.

##### Known insect vectors.

*Tomicusyunnanensis*, *T.minor*.

##### Known distribution.

Yunnan Province, China; Europe.

##### Specimens examined.

CHINA, Yunnan, from *Tomicusminor* and *T.yunnanensis* galleries in *Pinusyunnanensis*, Feb. 2017, Nov. 2016, HM Wang, CFCC 52611 = CXY 1866, CFCC 52612 = CXY 1865, CFCC 52613 = CXY 1868.

##### Note.

Our strains of *O.tingens* were identified based on phylogenetic affinities and morphological features. (cf. above under note for *O.aggregatum*.)

*Ophiostomatingens* has been reported from sapwood of various *Pinus* spp. (including *P.sylvestris*) infested by *T.minor*, *T.piniperda* and *Ipssexdentatus* in Europe (Francke-Grosmann 1952, Batra 1967, Jankowiak 2008). The species was recorded in Yunnan Province in China in 2017, associated with *T.minor* infesting *P.yunnanensis* (Pan et al. 2017).

#### Sporothrix macroconidia

Taxon classificationFungiOphiostomatalesOphiostomataceae

H. Wang, Q. Lu & Z. Zhang sp. n.

MB828886

Fig. 15

##### Etymology.

‘*macroconidia*’ (Latin), referring to the large conidia of this fungus.

##### Type.

**CHINA**, Yunnan, from *Tomicusyunnanensis* galleries in *Pinusyunnanensis*, Dec. 2016, collected by HM Wang, holotype CXY 1894, culture ex-holotype CFCC 52628 = CXY 1894.

##### Description.

Sexual form: unknown.

Asexual form: *Sporothrix*-like. *Conidiophores* semi-macronematous, mononematous; *conidiogenous cells* hyaline, simple or loosely branched, thin-walled, aseptate, bearing denticles forming a rachis (4.1–) 11.0–24.5 (–36.5) × (1.4–) 2.1–3.4 (–4.9) μm; *conidia* hyaline, cylindrical, ellipsoid to ovoid, 1-celled, smooth, (3.6–) 4.8–7.4 (–9.9) × (2.5–) 3.2–4.9 (–9.9) μm, solitarily or aggregating in slimy masses.

**Figure 15.**
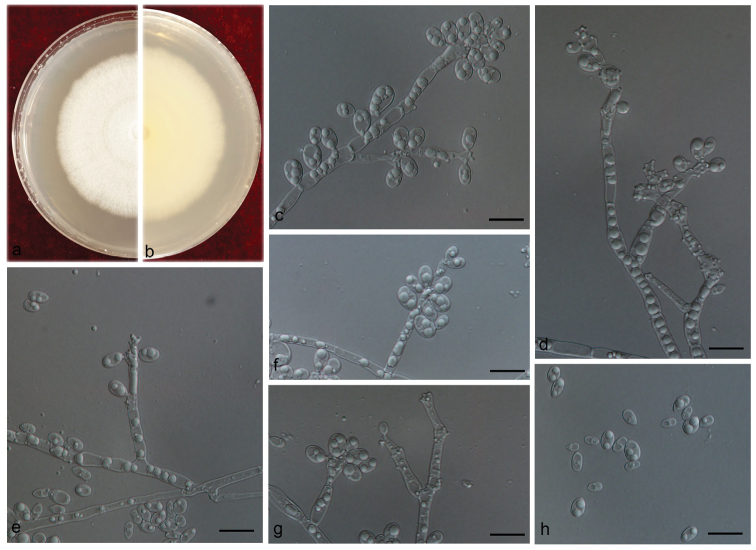
Morphological characters of *Sporothrixmacroconidia* sp. n. **A, B** Upper and reverse of cultures on 2% MEA 20 d after inoculation **C–H** conidiogenous cells of *Sporothrix*-like asexual state and conidia. Scale bars: 10 μm (**C–H)**.

##### Culture characteristics.

Colonies on 2% MEA medium slow growing in the dark, reaching 34 mm in diam. in 8 days at 25 °C, growth rate up to 5 mm/day at the fastest; colony margin smooth. Hyphae appressed to flocculose, white; reverse hyaline to pale yellowish. Optimal growth temperature 25 °C, little growth at 5 °C and 35 °C.

##### Known substrates and hosts.

Galleries of *Tomicusyunnanensis* and *T.brevipilosus* in *Pinusyunnanensis* and *P.kesiya*.

##### Known insect vectors.

*Tomicusyunnanensis*, *T.brevipilosus*.

##### Known distribution.

Yunnan Province, China.

##### Additional specimens examined.

CHINA, Yunnan, from *Tomicusbrevipilosus* galleries in *Pinuskesiya*, Dec. 2016, Jan. 2017, HM Wang, CFCC 52629 = CXY 1895, CFCC 52630 = CXY 1896.

##### Note.

*Sporothrixmacroconidia* is closely related to *O.valdivianum*, *S.bragantina*, *S.brunneoviolacea* and *S.fumea* in phylogenetic analyses inferred from LSU, ITS, *TUB2* and *CAL* DNA sequence data. It differs from these species by its conidia, which are larger than those of the other four species, mostly 4.8–7.4 × 3.2–4.9 μm and 4–6 × 2 μm in *O.valdivianum* (Butin and Aquilar 1984), 4–6 × 2–2.5 μm in *S.bragantina* (Pfenning and Oberwinkler 1993), 3–7 × 1.5–3 μm in *S.brunneoviolacea* (Madrid et al. 2010) and 1.5–2.0 × 0.5–1.0 μm in *S.fumea* (Nkuekam et al. 2012). In addition, a sexual state was observed *in vitro* for *O.valdivianum*, *S.bragantina* and *S.fumea*, which was not observed in *S.macroconidia* and *S.brunneoviolacea*.

*Sporothrixmacroconidia* was found associated with *T.yunnanensis* infesting *P.yunnanensis* and with *T.brevipilosus* infesting *P.kesiya*. The other four similar species have very different ecology and known geographic distributions. *Sporothrixfumea* was isolated from *Eucalyptuscloeziana* infested by *Phoracantha* beetles in South Africa (Nkuekam et al. 2012), whereas *O.valdivianum*, *S.bragantina* and *S.brunneoviolacea* were obtained from soil or *Nothofagus* in Europe and South America (Butin and Aquilar 1984, Pfenning and Oberwinkler 1993, Madrid et al. 2010).

#### Sporothrix pseudoabietina

Taxon classificationFungiOphiostomatalesOphiostomataceae

H. Wang, Q. Lu & Z. Zhang sp. n.

MB828887

Fig. 16

##### Etymology.

‘*pseudoabietina*’ (Latin), referring to the phylogenetic affinities to *S.abietina*.

##### Type.

**CHINA**, Yunnan, from *T.minor* galleries in *P.yunnanensis*, Apr. 2017, HM Wang, holotype CXY 1937, culture ex-holotype CFCC 52626 = CXY 1937.

##### Description.

Sexual form perithecial: on 2% MEA, *perithecia* superficial or partially immersed, with a globose base extending into a cylindrical neck, often terminated by ostiolar hyphae; bases (85–) 110–152 (–168) μm diam., black, the outer layer with dark brown hyphal ornamentation; apical neck mild to dark brown at the base, pale brown to pale yellow or hyaline toward the apex, straight or slightly curved, (172–) 560–985 (–1039) μm long, (37–) 41–62 (–78) μm wide at the base, (9.3–) 12.5–17.5 (–20) μm wide at the apex; *ostiolar* hyphae numerous, hyaline, divergent, (19.5–) 21.5–38.0 (–43) μm long; *asci* not seen; *ascospores* hyaline, 1-celled, orange-shaped in lateral view, ellipsoid in face view, circular in polar view, (2.9 –) 3.4–4.4 (–5.3) × (0.8–) 1.0–1.5 (–1.9) μm, without mucilaginous sheath.

Asexual form: *Sporothrix*-like. *Conidiophores* semi-macronematous to mononematous; *conidiogenous cells* hyaline, simple or loosely branched, smooth, bearing denticles disposed in a dense rachis (16.0–) 20.5–30.5 (–34.5) × (1.2–) 1.6–2.0 (–2.3) μm; *conidia* 1-celled, clavate, ellipsoid to ovoid, hyaline, (3.0–) 4.0–7.0 (–9.0) × (1.0–) 1.1–3.1 (–4.8) μm.

**Figure 16.**
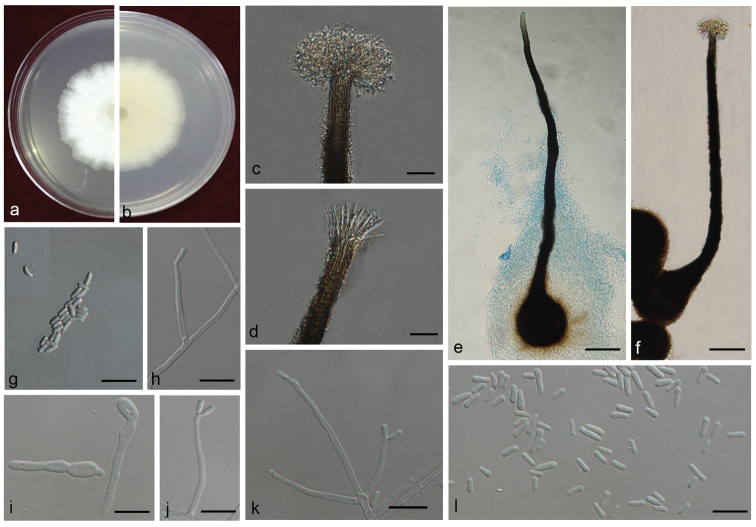
Morphological characters of *Sporothrixpseudoabietina* sp. n. **A, B** upper and reverse of cultures on 2% MEA 20 d after inoculation **C, D** ostiolar hyphae present **E, F** perithecium **G** ascospores of sexual state **H–I** conidiogenous cells of *Sporothrix*-like asexual state and conidia. Scale bars: 20 μm (**C, D)**; 50 μm (**E, F)**; 10 μm (**G–I)**.

##### Culture characteristics.

Colonies on 2% MEA slow growing in the dark, reaching 23 mm in diam. in 8 days at 25 °C, growth rate up to 2.5 mm/day at the fastest; colony margin smooth. Hyphae appressed to flocculose or floccose, white; reverse hyaline to pale yellowish. Optimal growth temperature 25 °C; very slow growth at 35 °C; no growth at 5 °C.

##### Known substrate and hosts.

Galleries of *Tomicusyunnanensis* and *T.minor* in *Pinusyunnanensis*.

##### Known insect vectors.

*Tomicusyunnanensis*, *T.minor*.

##### Known distribution.

Yunnan Province, China.

##### Additional specimen examined.

CHINA, Yunnan, *Tomicusminor* galleries in *Pinusyunnanensis*, Apr. 2017, HM Wang, CFCC 52627 = CXY 1938.

##### Note.

*Sporothrixpseudoabietina* is characterised by a perithecial sexual form and a *Sporothrix*-like asexual state. Multiple phylogenetic inferences (LSU, ITS, *TUB2* and *CAL*) showed that *S.pseudoabietina* belonged to the *S.gossypina* complex, in which it is closely related to *S.abietina*. However, it can be distinguished from this species, based on both morphological and physiological features. The conidia of *S.pseudoabietina* (4.0–7.0 × 1.1–3.1 μm) are wider than those of *S.abietina* (4–7.5 × 1–2 μm) (Marmolejo and Butin 1990). Perithecia are known from *S.abietina* but only on natural substrates and not *in vitro* on artificial media, contrary to those from *S.pseudoabietina*. The perithecial neck in *S.pseudoabietina* is much longer than that of *S.abietina*, *viz.* mostly 560–985 μm and 450–650 μm, respectively. Ostiolar hyphae of *S.abietina* and *S.pseudoabietina* also differ in number, numerous *vs* 7–10 and size, mostly 13–19 μm and in *S.pseudoabietina* 21.5–38.0 μm (Fig. 11c, d). In addition, no growth of *S.abietina* was observed at 35 °C, but *S.pseudoabietina* can grow at 35 °C.

The hosts and geographic distributions of *S.pseudoabietina* and *S.abietina* are also very different. *Sporothrixpseudoabietina* was found associated with *T.minor* and *T.yunnanensis* infecting *P.yunnanensis*, whereas *S.abietina* was reported from *Abiesvejari* attacked by *Pseudohylesinus* sp. in Mexico (Marmolejo and Butin 1990).

## Discussion

In this study, 772 strains of ophiostomatoid fungi were isolated from galleries and adults of three pine shoot beetles, *T.brevipilosus*, *T.minor* and *T.yunnanensis*, inhabiting *P.yunnanensis* and *P.kesiya* in forests in Yunnan Province, south-western China. Multiple phylogenetic analyses and morphological features allowed the identification of 11 species from 5 genera. Six species corresponded to known taxa (*E.vermicola*, *L.yunnanense*, *O.brevipilosi*, *O.canum*, *O.minus* and *O.tingens*), whereas four species are proposed as new, *Gra.anningense*, *O.aggregatum*, *S.pseudoabietina* and *S.macroconidia*. A single strain remained unnamed.

The global ophiostomatoid fungal communities, associated with these three *Tomicus* species in pine forest, were dominated by far by three species, which are, in decreasing order of isolates, *O.canum*, *O.brevipilosi* and *O.minus*. Furthermore, these three ophiostomatoid species are not equally associated with the three *Tomicus* species but show variable degrees of preference or specificity.

Overall, *O.canum* was the most frequently isolated species in our study (253 out of the 772 strains). It was preferably (79.4% of the *O.canum* strains) isolated from galleries and adults of *T.minor*, infesting both *P.yunnanensis* and *P.kesiya* (Table 3) and dominated the ophiostomatoid community associated with this beetle (81.4%, 201 strains of *O.canum* out of 247 strains in the community, Table 3).

This is the first report of this species in China. It was previously reported in eastern Asia but only in Japan (Masuya et al. 1999). *Ophiostomacanum* was also shown to be the dominant species associated with *T.minor*, both in Europe and Japan (Masuya et al. 1999, Jankowiak 2008). In addition, this species was found in association with other bark beetles in Finland and Russia, e.g. *Hylastesbrunneus*, *Hylurgopspalliatus*, *Ipstypographus*, *Pityogeneschalcographus* and *Trypodendronlineatum* (Linnakoski et al. 2010). The close association between *O.canum* and *T.minor* appears stable over an extensive geographical distribution and tree host range, indicating likely intimate relationships.

*Ophiostomabrevipilosi* represented the second most frequently isolated species in our survey (224 out of 772 strains), occurring exclusively in galleries and adults of *T.brevipilosus*, dominating this beetle’s ophiostomatoid community (98.2%, 224 strains of *O.brevipilosi* out of 228 strains in the community, Table 3). The occurrence or fitness of *O.brevipilosi* is therefore strongly linked to the presence of *T.brevipilosus*.

*Ophiostomabrevipilosi* was described originally from Yunnan, based on six strains, all isolated from *T.brevipilosus* (Chang et al. 2017). It belongs to the recently defined O.clavatum complex (Linnakoski et al. 2016). It is only known from this area of south-western China.

*Ophiostomaminus* was the third most frequently isolated species overall (197 strains out of 772), occurring exclusively in galleries and adults of *T.yunnanensis* infesting *P.yunnanensis*, dominating this beetle ophiostomatoid community (66.3%, 197 strains of *O.minus* out of 297 strains in the community, Table 3).

*Ophiostomaminus*, first reported as a blue-stain agent in Europe (Munch 1907), is a widely distributed species, also recorded from North America and East Asia (Japan and China) (Hedgcock 1906, Gorton and Webber 2000, Gorton et al. 2004, Lu et al. 2009, Linnakoski et al. 2010). It infests various pines and is transported by various bark beetles. This species was predominantly associated with *T.piniperda* in Europe (Jankowiak 2006) and Japan (Masuya et al. 1999) and with the southern pine beetle, *Dendroctonusfrontalis*, in the southern states of the USA (Klepzig 1998, Gorton and Webber 2000, Gorton et al. 2004).

*Ophiostomaminus* was deemed to have two allopatric populations, *viz.* a North American and a Eurasian population (Gorton et al. 2004). In ITS/*TUB2* phylogenetic inferences, the North American and Eurasian populations of *O.minus* were resolved as two closely related clades (Gorton et al. 2004, Lu et al. 2009). ITS and *TUB2*-based phylogenetic inferences (Fig. 7c, d) also resolved our strains as a third distinct clade, which could thus be interpreted as a third allopatric population. The question of translating these populations into a Linnaean taxonomic rank, however, remains open.

**Figure 17.**
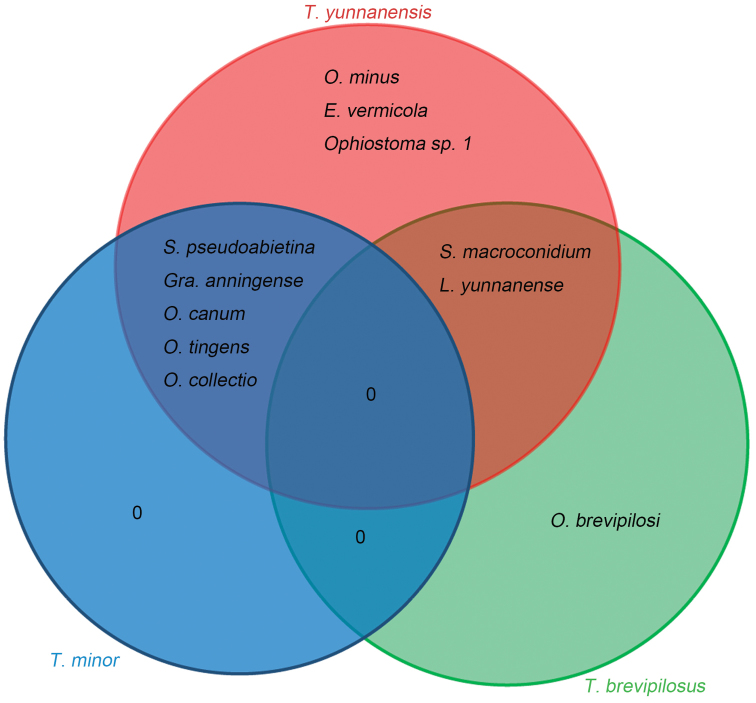
Venn diagram showing overlaps of the ophiostomatoid fungal communities associated with three pine shoot beetles.

*Tomicusyunnanensis* galleries and adult beetles harboured the highest diversity of ophiostomatoid fungi; ten of the 11 species identified were isolated from galleries and adults of this beetle. Three species were exclusively found with this beetle (*O.minus*, *E.vermicola*, *Ophiostoma* sp. 1). By comparison, galleries and adults of *T.minor* and of *T.brevipilosus* yielded less species; five species were isolated from *T.minor*, none of which was associated exclusively with this beetle and three species from *T.brevipilosus*, of which one was exclusive, *O.brevipilosi*. Five species are shared by both *T.yunnanensis* and *T.minor* and two species by both *T.yunnanensis* and *T.brevipilosus*, but none by *T.minor* and *T.brevipilosus* and also none by all three pine shoot beetles (Table 3, Fig. 17).

**Table 3.** Strain numbers of various ophiostomatoid fungi obtained from three *Tomicus* spp. and their galleries collected in Yunnan Province.

**Group**	**Fungi species**	*** Tomicus yunnanensis ***	*** T. minor ***	*** T. brevipilosus ***	**Total no. strains\samples**
A	* Ophiostoma brevipilosi *	0	0	224	224
B	* O. canum *	52	201	0	253
C	* O. minus *	197	0	0	197
D	* O. tingens *	4	26	0	30
E	* O. aggregatum *	3	2	0	5
F	*Ophiostoma* sp. 1	1	0	0	1
G	* Leptographium yunnanense *	30	0	2	32
H	* Esteya vermicola *	1	0	0	1
I	* Sporothrix pseudoabietina *	4	15	0	19
J	* S. macroconidia *	1	0	2	3
K	* Graphilbum anningense *	4	3	0	7
	Total no. strains	297	247	228	772
	Total no. samples	455	324	339	1118

The ectosymbiosis between bark beetles and fungi is widespread and diverse. Some fungi are highly specific and associated with a single beetle species, forming a ‘species-specific association’ (Six and Paine 1999, Six 2012), while others can be associated with many vectors (Kostovcik et al. 2014). The species-specific associations include, for instance, *Ipstypographus* and *Endoconidiophorapolonica*, *I.cembrae* and *End.laricicola* (Harrington et al. 2002) or *I.subelongatus* and *End.fujiensis* (Marin et al. 2005, Meng et al. 2015). The present study showed that species-specific associations might occur with various sympatric beetles that share the same niche. The association of *T.brevipilosus* and *O.brevipilosi* seems to be species-specific in the pine forest of Yunnan, where both taxa are, so far, endemic. In the pine forest of Yunnan, the Chinese ‘population’ of *O.minus* is also specifically associated with *T.yunnanensis*, whereas the two other *O.minus* ‘populations’ are associated, at least preferably, with *Dendroctonusfrontalis* and *T.piniperda* (Gorton et al. 2004, Jankowiak 2006). The genetically distinct ‘populations’ might originate from both the allopatric distribution and vector specificity and both factors could support recognition of three distinct taxa. In the pine forest of Yunnan, the association of *O.canum* with *T.minor* is preferential but not exclusive.

Up to now, no data have been provided proving the pathogenicity of these ophiostomatoid species to both indigenous pines, except for *L.yunnanense* (Liao and Ye 2004, Gao et al. 2017). Pathogenicity tests have been done by artificial inoculation of the dominant species into seedlings of the two pines. The results preliminarily showed that the virulence of *O.minus* and *O.brevipilosi* was significantly stronger than that of *O.canum*. This is similar to the relative aggressive nature of the three *Tomicus* species. Thus, we suspect there might be some link between beetle aggression and fungus virulence (Christiansen et al. 1987, Kirisits 2004).

## Conclusions

This study provides evidence for the diversity of ophiostomatoid fungi associated with *T.yunnanensis*, *T.minor* and *T.brevipilosus* in Yunnan pine forest in south-western China. Eleven species were identified, of which four were new to science. The diversity is the highest in the galleries and adults of *T.yunnanensis* and the poorest in the galleries and adults of *T.brevipilosus*.

Three species, namely *O.brevipilosi*, *O.canum* and *O.minus*, dominate the ophiostomatoid communities; each is associated predominantly with one species of *Tomicus*, namely *T.brevipilosus*, *T.minor* and *T.yunnanensis*, respectively. In this regard, this study has revealed differential associations between beetles living sympatrically, concomitantly or sequentially, in the same ecological niche, which indicates a certain level of specificity of the relationships between the fungi and the beetles. However, the parameters behind these (partial) species-specific relationships remain unknown.

Increased study of the biodiversity, biogeography and ecology of ophiostomatoid fungi in China, in particular of those associated with *Tomicus* spp., would facilitate comparison with well-known species associated with other *Tomicus* spp. in other neighbouring or distant geographical areas, e.g. in European countries, Japan and Korea and allow a better understanding of the occurrence and mechanisms behind the outbreak of infections, enabling the development of effective management methods to alleviate the subsequent plant losses.

## Supplementary Material

XML Treatment for Esteya vermicola

XML Treatment for Graphilbum anningense

XML Treatment for Leptographium yunnanense

XML Treatment for Ophiostoma aggregatum

XML Treatment for Ophiostoma tingens

XML Treatment for Sporothrix macroconidia

XML Treatment for Sporothrix pseudoabietina
